# Targeted memory reactivation during sleep improves emotional memory modulation following imagery rescripting

**DOI:** 10.1038/s41398-024-03192-4

**Published:** 2024-12-18

**Authors:** Dominique Recher, Judith Rohde, Giulia Da Poian, Mirka Henninger, Luzius Brogli, Reto Huber, Walter Karlen, Caroline Lustenberger, Birgit Kleim

**Affiliations:** 1 Experimental Psychopathology and Psychotherapy, Department of Psychology, University of Zurich, Zurich, Switzerland; 2 Department of Adult Psychiatry and Psychotherapy, Psychiatric University Clinic Zurich and University of Zurich, Zurich, Switzerland; 3 Sensory-Motor System Lab, Institute of Robotics and Intelligent Systems, Department of Health Sciences and Technology, ETH Zurich, Zurich, Switzerland; 4 Psychological Methods, Evaluation and Statistics, Department of Psychology, University of Zurich, Zurich, Switzerland; 5 Statistics and Data Science, Department of Psychology, University of Basel, Basel, Switzerland; 6 Institute of Biomedical Engineering, Faculty of Engineering, Computer Science and Psychology, Ulm University, Ulm, Germany; 7 Cognitive Neuroscience of Memory and Consciousness, Department of Psychology, University of Bern, Bern, Switzerland; 8 Child Development Center, University Children’s Hospital Zurich, University of Zurich, Zurich, Switzerland; 9 Department of Child and Adolescent Psychiatry and Psychotherapy, Psychiatric Hospital, University of Zurich, Zurich, Switzerland; 10 Neuroscience Center Zurich, University of Zurich, Zurich, Switzerland; 11 Neural Control of Movement Lab, Institute of Human Movement Sciences and Sport, Department of Health Sciences and Technology, ETH Zurich, Zurich, Switzerland

**Keywords:** Human behaviour, Long-term memory, Physiology

## Abstract

Targeted Memory Reactivation (TMR) during sleep benefits memory integration and consolidation. In this pre-registered study, we investigated the effects of TMR applied during non-rapid eye movement (NREM) sleep following modulation and updating of aversive autobiographical memories using imagery rescripting (ImR). During 2–5 nights postImR, 80 healthy participants were repeatedly presented with either idiosyncratic words from an ImR updated memory during sleep (experimental group) or with no or neutral words (control groups) using a wearable EEG device (Mobile Health Systems Lab-Sleepband, MHSL-SB) [1] implementing a close-loop cueing procedure. Multivariate analysis were conducted to assess change score trajectories in five key emotional memory characteristics (positive and negative valence, emotional distress, arousal, and vividness) across assessments (timepoints, *t*) and between the study groups (TMR condition). While ImR showed significant effects on all memory characteristics (*d* = 0.76–1.66), there were significant additional improvements in the experimental group. Memories were significantly less vivid and afflicted with less emotional distress and arousal following ImR-words cueing. TMR during sleep in individuals’ homes was feasible and further improved some ImR’s adaptive memory effects. If replicated in clinical samples, TMR may be utilized to augment the effects of ImR and other clinical memory modulation procedures and create personalized treatment options. Such advances in emotional memory treatments are direly needed, as aversive memories are a salient feature across mental disorders, such as post-traumatic stress disorder (PTSD).

## Introduction

Aversive emotional memories are a salient feature across mental disorders such as PTSD, anxiety, and depression, and they can be highly disruptive, e.g., in the form of intrusive images or resulting negative schemas and memory biases [2, 3]. Evidence-based treatments for such disorders, e.g., cognitive behavioral therapy (CBT), aim to modulate such distressing memories, e.g., through updating and formation of more adaptive memory traces, which is a crucial process in driving clinical symptom change [4, 5]. Imagery rescripting (ImR) is one such therapeutic technique and established clinical procedure [6, 7]. It prompts patients to relive and rescript and thereby to modulate distressing experiences and afflicted memories [8]. ImR is a prime example of learning during which previously consolidated memories are retrieved and, through activation, transferred into a labile state. In this temporary state, memories become receptive to modulation and integration of newly acquired experiences, such as adaptive information and emotional processing experienced during treatment [9]. Although not all working mechanisms are yet fully understood [10], effects of such techniques presumably depend on the re-stabilisation of these adaptively updated memory traces in subsequent phases of reconsolidation to solidify treatment effects [11]. According to the theory of active system consolidation, such re-consolidation processes occur preferably offline during sleep [12, 13]. Specifically, sleep-dependent memory consolidation is thought to rely on spontaneous, repeated reactivation of neuronal representations from previous awake encoding episodes, especially during slow waves (SWs) occurring during NREM sleep [14]. Through this offline reactivation, initially labile memory traces encoded or updated while awake are gradually reorganized and become integrated into neocortical networks of pre-existing long-term memories [15, 16], transforming them into stable and more persistent memory representations [17].

TMR is a technique to non-invasively manipulate and enhance offline re-consolidation during sleep. In TMR procedures, sensory cues are presented during awake learning, and these cues are later presented again during sleep, which presumably biases spontaneous offline memory reactivation towards reactivation and consolidation of the specific memories associated with the sensory cue [18]. TMR thus opens the possibility to non-invasively reactivate a selected subset of specific memories and facilitate integration and re-stabilization of this memory within pre-existing memory traces [19]. While the first meta-analysis suggests that TMR during slow-wave sleep (SWS) and N2 sleep improves memory performance for declarative and procedural memories [20], few studies have examined TMR-associated emotional memory consolidation, and results are mixed [21–27], although some promising findings exist. TMR during NREM sleep significantly modulated conditioned fear memories [28, 29], changed the interpretation of ambiguous scenes [30, 31], and enhanced adaptive learning and schema up-dating [32, 33]. Despite frequent calls to investigate the potential of TMR to enhance clinical applications [34–37], studies applying TMR to enhance reconsolidation of adaptively altered idiosyncratic memories post-treatment are scarce and results are inconclusive, e.g., in the context of TMR’s potential to enhance exposure therapy [38, 39]. However, such methods to enhance psychotherapeutic treatment are direly needed, as approximately half of patients do not benefit from first-line treatments [40–43]. While most TMR studies have been conducted in the sleep laboratory during single nights, ambulatory TMR settings appear particularly promising for clinical implementation in patient’s familiar sleep environment.

Here, we investigated the effects of TMR on offline emotional memory reactivation and re-consolidation following ImR memory modulation of socially aversive memories. Aversive autobiographical memories, and particularly memories of adverse interpersonal experiences (e.g., bullying, rejection in a relationship), are a prevalent and distressing feature and independent predictor across mental disorders, and are a key target in treatment [3, 44]. During 2–5 experimental nights after the ImR, participants received different TMR conditions in NREM sleep using a wearable EEG device [1]. Participants in the experimental group (EG) received TMR with words from the ImR updated memory compared to the control groups (CGs), who received no words or cueing with neutral words. We hypothesized that participants in the EG show better ImR effects, as evidenced by additional improvements in emotional memory characteristics in the EG compared to the CG. Exploratorily, we investigated effects of cueing dose and whether two (EG-2) vs. five (EG-1) ImR cueing nights are associated with more additional improvements in memory characteristics.

## Methods

### Participants

Participants were 80 young, healthy individuals (see Table 1) who reported a memory of a distressing socially aversive life event (e.g., bullying experience) that they still experience in the form of distressing and intrusive memories. They were recruited via flyers distributed through mailing lists of various university institutes of Zurich, as well as via social media. Participants were eligible if their memory concerned a socially aversive, but non-traumatic event that had occurred at least half a year ago and was still associated with increased distress. They were required to speak fluent German and to commit to maintain a regular sleep-wake rhythm based on their personal sleep habits and to refrain from deviations in their usual daily routines that might interfere with sleep during the days following a night with the MHSL-SB (see online pre-registration for more details). Participants were excluded if they received current psychiatric/psychotherapeutic treatment and/or had a current psychiatric diagnosis, showed signs of impaired or disturbed sleep and drug or alcohol abuse, if they were taking psychotropic medications, including sleep-promoting medications, and if they worked night shifts.

**Table 1:** Participant characteristics (*N* = 80) and memory features at Baseline (*t*_0_).

Variable	*n* (%)	*M*	*SD*	Range
**Sex**				
Female	58 (72.5)			
Male	22 (27.5)			
**Age**		23.0	3.68	18–35
**Racial/ethnic identification**				
Swiss	59 (73.75)			
Non-Swiss European	12 (15.0)			
Non-European	3 (3.75)			
Mixed (Swiss-European)	2 (2.5)			
Mixed (Swiss-Non-European)	4 (5.0)			
**Highest completed education**				
High school	58 (72.5)			
Apprenticeship	6 (7.5)			
University	16 (20.0)			
**Depressive, anxiety and sleep symptoms**				
BDI-II		7.03	5.95	0–29
BAI		8.38	7.02	0–34
PSQI		4.08	1.75	1–9
**Emotional memory features**				
Age at the time of the event		16.01	6.09	6–34
Years since memory		6.96	5.69	1–27
Extent to which memory influences self-perception^*1^		6.38	2.08	2–10
Number of participants reporting intrusions^*2^	41 (51.25)			
Number of intrusions pre-ImR^*2^	71 (54.20)			
Number of intrusions post-ImR^*2^	60 (45.80)			

*BDI-II* Beck Depression Inventory-II, clinical cut-off = 14 [93]; *BAI* Beck Anxiety Inventory, clinical cut-off = 16 [94]; *PSQI* Pittsburgh Sleep Questionnaire, clinical cut-off = 5 [95]; *ImR* Imagery Rescripting; *M* Mean. The four study groups did not differ significantly in their characteristics at baseline (see Supplementary Fig. 2 and Tables 1 and 2). ^*1^Rating of the item “to what extent has the memory influenced how you perceive yourself today” on a Likert-type scale ranging from 1 (not at all) to 10 (very strongly). ^*2^ Intrusion diary assessing intrusions in daily life via study app within both 7 days pre- and post-ImR. The low number of participants reporting intrusions in both time intervals pre- and post-ImR (*n* = 11), did not allow for valid group comparison regarding ImR or TMR effects.

To reach the pre-registered target sample of *n* = 80, a total of 90 eligible participants were randomized, see the CONSORT flow diagram in the Supplementary Fig. 1. Of these, we excluded ten participants mostly for technical reasons, such as problems with the MHSL-SB that prevented valid EEG recording and valid cueing during at least one of the two experimental nights. All participants for whom at least one valid EEG night recording and valid cueing routine was achieved were retained in the final sample. 40 participants each were randomly assigned to the two experimental groups (EG-1 and EG-2) and to the control groups (CG-1 and CG-2). The pre-registered number of participants should have been sufficient to detect a small effect size (*f* = 0.15) with 95% power according to an approximated G*Power analyzes applying a mixed ANOVA for two groups over 6 timepoints [45], which, however, can only serve as a proxy to our complex multivariate multilevel analyzes inherent to our design.

### Design and procedures

We pre-registered this double-blind RCT and our hypotheses, including detailed information on the procedures (https://osf.io/dsej7). This study obtained ethical approval by the Ethics Committee of the Faculty of Arts at the University of Zurich (approval number 19.10.9) and was conducted in accordance with the Declaration of Helsinki and the Good Clinical Practice (GCP) guidelines. All participants provided written informed consent before participation. Participants received either financial compensation or course credits.

**Fig. 1:**
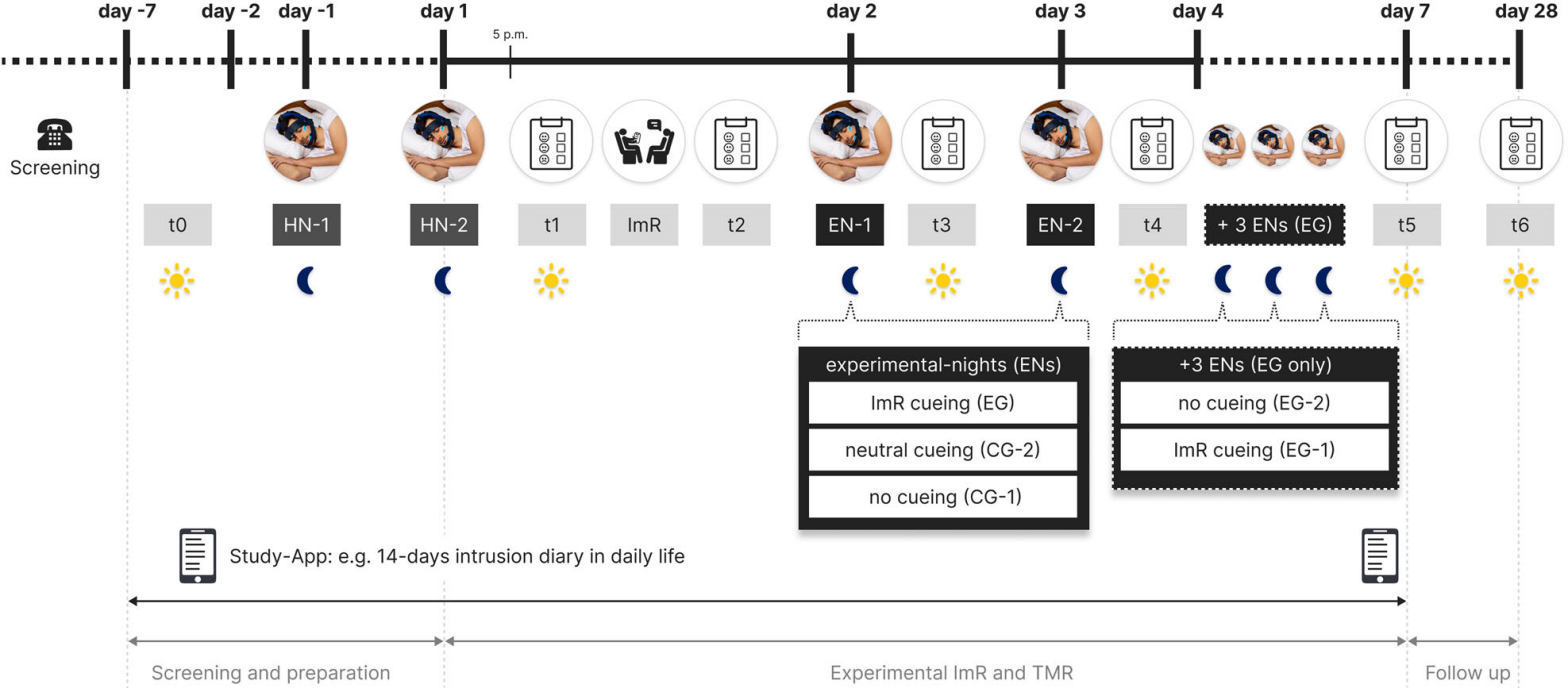
Study design. Visualization of the 5-week experimental paradigm, a between-person double-blind randomized controlled trial. **(I) screening** **and preparation (days −7 to 1):** Participants underwent a screening, and eligible participants attended the first appointment (t0, scheduled 2–5 days before the ImR memory modulation). Two nights before the ImR intervention, participants completed two habituation nights (HN-1 and HN-2) with the wearable EEG. Participants completed an app-based intrusion diary during seven days pre-ImR. **(II) Experimental ImR and TMR (days 1 to 7):** ImR took place in the evening of day 1, including a pre-ImR (t1) and post-ImR (t2) script driven imagery procedure to assess memory characteristics. Participants then were randomized to cueing conditions and completed two experimental nights (EN-1 and EN-2): cueing with words from the ImR updated memory (experimental group, EG) vs. cueing with neutral words (control group 2, CG-2) or no cueing (control group 1, CG-1). The script-driven imagery procedure to assess memory characteristics was repeated online via videocall each morning 30 min after the habitual wakening time after EN-1 (t3) and EN-2 (t4). Participants in the EG were then asked to complete three additional nights with the MHSL-SB between day 3 and day 7 with participants in EG-1 continue to receive ImR words cueing, whereases participants in EG-2 received three additional sham-EEG nights (without ImR words cueing). All participants continued with the app-based intrusion diary within seven days post-ImR. **(II) Follow-up (days 7 to 28):** Script-driven imagery to assess emotional memory characteristics at 1-week (t5, in-lab) and 1-month (t6, online via video call) post-ImR.

Participants in our study were randomly assigned to four conditions (EG-1: *n* = 22, EG-2: *n* = 18, CG-1: *n* = 20, CG-2: *n* = 20; see CONSORT flow diagram in the Supplementary Fig. 1) and the study was double-blinded. Neither the experimenter nor the participant knew about the cueing protocol assignment (between-person variable). Participants completed a 5-week experimental between-person study protocol including six study appointments, two electroencephalography (EEG) habituation-nights and 2–5 EEG experimental nights using the MHSL-SB [1, 46], see Fig. 1. The four study groups received distinct TMR cueing protocols during the 2–5 experimental nights.

#### Screening and preparation

Participants completed an online questionnaire that included a list of socially-aversive events and associated distresses, yet the events did not meet the DSM-5 event criterion for PTSD [47, 48]. A telephone screening verified inclusion and exclusion criteria. Eligible and interested participants were then instructed to install a study app on their smartphone and to use it for seven days before the memory modulation. At the first appointment (t0), participants completed questionnaires on demographic and clinical variables and an imagination training on neutral content. In addition, a neutral autobiographical memory was collected from all participants, which later served for the selection of neutral cues in the CG-2. Finally, participants were trained on how to use the MHSL-SB at home. They completed two habituation nights with the MHSL-SB in order to get used to sleeping with the MHSL-SB at home prior to the ImR memory modulation.

#### ImR and TMR

In the evening after the second habituation night, participants received the ImR memory modulation intervention. Pre- (baseline, t1) and post-ImR intervention (t2), memory characteristics were assessed using a script-driven imagination procedure [49, 50]. Participants then left the laboratory and completed two experimental nights with the MHSL-SB at home, receiving a TMR cueing protocol according to the randomized study group assignment. Participants were instructed to spend a quiet and relaxed evening within their usual routines and to not talk to anyone about the memory and/or the intervention. Prior to the two experimental nights, participants completed an activity and mood log via Ecological Momentary Assessment (EMA) implemented in the study app, showing that they spent their time on routine daily tasks and thus seem to adhere to the study’s instructions. In the morning after each of the two experimental nights, an online video call was held 30 min after the habitual wake-up time and memory characteristics of the emotional and neutral memory were assessed again using the script-driven imagination (t3 and t4). Following t4, participants were requested by an unblinded investigator whether they should complete three additional nights with the MHSL-SB (EG) or no additional nights (CG). They were also instructed to complete the study app and the intrusion diary for seven more days post ImR memory modulation.

#### Follow-up (FU)

Questionnaires and the script-driven imagery procedure to assess memory characteristics were repeated at two FU assessments at one-week (FU-1, t5) and at one-month (FU-2, t6) after the ImR memory intervention. In addition, the clinical questionnaires applied at t0 were re-administered at both follow-up assessments.

#### ImR memory intervention and TMR cueing words selection

We used an established clinical ImR procedure adapted from Wild and Clark [51]. A cognitive restructuring of the distressing negative belief associated with the memory was first carried out. Subsequently, the memory was processed in three steps. First, participants relived the event from the perspective of their younger self at the time of the event and described all thoughts and feelings. Second, their current-self observed the younger-self reliving the situation. Third, the current-self intervened in the imagined scene supporting the younger-self in confronting perpetrator(s) and providing emotional comfort. Changes in beliefs and new insights were taken up and integrated into the memory. At the end, a positive closing image was initiated portraying the personally most significant image and meaningful changes of the whole scene, aiming to end the process with a sense of completion, including significant emotions, cognitions, sensations and behaviors. The memory interventions were delivered by five trained master’s level psychology students (experimenters). In addition, the ImR-sessions were audiotaped and supervised weekly by a trained psychologist or psychiatrist (DR and JR). Following the intervention, the experimenter created two lists with a pre-selection of ten words each according to a standardized procedure. One list contained ten words from the ImR-updated emotional memory (e.g., idiosyncratic keywords, i.e., positive emotions, sensations, cognitions, or behavior from the positive closing image or ImR phase three, where most of the meaningful changes occurred). The other list contained ten words representing the neutral memory (e.g., specific memories of a neutral routine activity, such as this morning’s breakfast, and the keywords used included descriptions of objects, the environment, etc.). Participants were then instructed to identify the ten most representative words for each of the two memories, with the emotional memory words specifically representing the modified version of the emotional memory, e.g., significant insights and meaningful changes and associated feelings and thoughts etc. To assist, the participants were provided with the two-word lists and were instructed to reflect on how the words represented each memory and were allowed to freely exchange the words. This procedure was intended to strengthen the associations between the chosen cues and the respective memory (we provide the standardized templates for the selection of keywords and the procedures on OSF). Examples of such highly individualized words for the up-dated emotional memory were, e.g., “hug” (as some participants in the closing image imagined their current self-hugging their younger self), “soothe”, “I’m okay”, “calm”, “happy”. The same procedure was then repeated for the neutral memory. The word cues were then recorded by the respective experimenter and edited using Audacity® version 2.3.3, including normalizing the audio amplitude and cropping the cues, which were then used as TMR cues.

#### Wearable EEG (MHSL-SB) and TMR cueing-protocol

Biosignal recordings and TMR word cueing was performed using the MHSL-SB, a wearable and configurable system for real-time EEG sleep recording and processing [1, 46]. The MHSL-SB uses a single EEG signal (Fpz-A2) for real-time processing, while two additional EOG and a submental EMG signal were recorded for post-processing purposes. Self-adhesive single-use electrodes (Ambu BlueSensor N electrode, DK) enabled self-application. Biosignals were sampled at 250 Hz using a 24-bit analogue-to-digital converter equipped with an on-chip anti-aliasing filter. The raw bio-signals, including AC and DC components, were stored on an SD card and forwarded to the embedded real-time algorithms utilized for the words-cueing. As in previous studies employing the established MHSL-SB [46, 52], the quality of the EEG data recordings was high (see examples of raw EEG traces in the Supplementary Fig. 7). To enable precise words-cueing during SWs rich NREM sleep, the MHSL-SB implemented a set of feedback-controlled stimulation algorithms. The single EEG signal was processed to remove power line noise (first-order notch filter, 50 Hz). Then, overall, a stimulation decision logic triggered the cueing-words-stimuli as soon as the conditions NREM sleep, SWs and low beta power were simultaneously met. A detailed description of the algorithms used is provided by Lustenberger et al. [46]. Under the conditions set by the algorithm, the 10 words were played in a block-pseudo-randomized order preferably during SWs, without time-locking the cueing to a specific SW-phase. An inter-stimulus-interval of 3.5 s between words was specified based on evidence that a critical time window after a cue is required for oscillations to occur that are putatively relevant to let the cueing specific effects unfold [53]. The initial volume of the auditory stimuli was set to 50 dB SPL. To prevent participants from being awakened, the algorithm adjusted the volume when signs of arousal were detected [46].

Participants in the EG (*n* = 40) were cued with words from the updated memory during SWs in both experimental nights. The CG (*n* = 40) was further divided into two sub-groups. Individuals in CG-1 (*n* = 20) received no cues during SWs while individuals in CG-2 (*n* = 20) were re-exposed to neutral words during SWs. CG-2 served to control for the fact that words were played during SWs. To investigate the exploratory research question of whether more cueing nights could further enhance the TMR effects on the key emotional memory characteristics, also the EG was sub-divided (for more details see CONSORT flow diagram in the Supplementary Fig. 1). Participants in EG-1 (*n* = 22) received three additional nights with emotional words cueing, while participants in EG-2 (*n* = 18) had three additional nights of EEG recordings without cueing. These three sham-EEG-nights without cueing served to control for possible expectancy effects between the two EG-groups.

### Measures

#### Memory characteristics (primary outcomes)

A standardized script-driven imagery procedure [49, 50] served to reactivate the memory, followed by an assessment of the memory characteristics. A 40–60 words script for each memory containing key elements of the memory was read out and participants were asked to form a clear picture of the event in their inner mind’s eye. Once affirmed, they were asked to relive the event as vividly as possible involving all their sensory perceptions and emotions for 30 s. Key memory characteristics were then assessed, i.e., positive and negative valence, arousal and vividness, all measured on a 10-point Likert-type scale ranging from 1 “not at all” to 10 “very strongly”. In addition, negative emotional distress was assessed using the Emotional Distress Inventory [54], evaluating 11 negative emotions associated with the memory (anxiety, guilt, shame, anger, helplessness, sad, loneliness, disgust, horror, shocked/frightened, resignation), each indexed on a 10-point Likert-type scale. With the exception of one missing set of emotional memory characteristics at t_3_ for one participant (CG-2) (due to problems with the online video portal), data on the emotional memory characteristics are complete.

#### Memory-related negative beliefs (secondary outcome)

Participants identified their most distressing memory-related belief and were asked how distressing it was on a Likert-type scale ranging from 1 (“not at all”) to 10 (“very strongly”). For two participants (EG-1 and CG-2) assessments of the distress associated with the negative beliefs at t_0_ and t_1_ were missing, thus these individuals were excluded from the negative belief related analysis.

#### Intrusions in daily life (secondary outcome)

Using the study app, intrusions were monitored for a two week period, exactly seven days each before and after the ImR memory modulation. The app was developed using the open-source software SEMA (Smartphone Ecological Momentary Assessment) [55] and included an intrusion diary in which participants were asked to record any intrusive memories of the targeted socially-aversive event in their daily lives.

#### Psychophysiological arousal (secondary outcome)

Heart rate (HR) was measured using the Empatica E4 wristband [56], which participants wore during all memory assessments in-lab, namely before (t_1_) and after the ImR intervention (t_2_), and at the one-week FU-1 (t_5_). HR data count was reduced due to technical artefacts and failures in a total of *n* = 13 recordings across all timepoints (8.2%) (see Supplementary Fig. 3).

### Data pre-processing

#### EEG sleep stage scoring

Automatic sleep stage scoring was performed using a deep learning classification algorithm with a similar design as introduced in [57]. To test the performance of the automatic sleep phase scoring algorithm, one expert rater scored manually a series of ten representative nights. The randomly drawn sample of *n* = 10 nights featured the same ratio in terms of age, sex, and time of measurement during the study period as the total sample. Inter-rater agreement between the deep learning based automatic sleep stage scoring algorithm and the manually scored sleep stages was adequate (Cohen’s kappa = 0.68; 95% CI: 0.66–0.69) (see Supplementary Fig. 8) [58, 59].

#### Heart rate (HR) response

During all in-lab memory assessments (t_1_, t_2_ and t_5_), markers were set on the E4 wristband for different parts of the study. To control for putative intra-individual differences in baseline HR across the three timepoints (e.g., due to circadian effects), HR change scores were calculated by subtracting a 90-s interval of the emotional from the neutral memory script-driven imagery procedure (including the instruction to slowly wander back in time, followed by the reading out of the memory script and the 30-s re-experience interval) for each time point (for a further description, see Supplementary Fig. 3).

### Statistical analyzes

Analyzes were conducted using R 4.0.2 [60]. Where necessary, the memory characteristic variables were transformed to meet distributional modelling assumptions (see Supplementary Fig. 6). We specified a two-level multivariate multilevel model for each of the five emotional memory characteristic (primary outcomes) and for the distress associated with the memory’s negative belief (secondary outcome) as the outcome variables, with measurement occasions on Level-1 being nested in participants on Level 2. To test the pre-registered hypothesized change trajectories and group differences in emotional memory characteristics between the four study groups (differing regarding the TMR cueing protocol applied), we conducted multivariate contrast analysis (see for example [61–63]). Eight contrasts were defined to test the pre-registered hypothesized expected change trajectories in emotional memory characteristics across the six memory assessments (time points) between the four study groups (TMR condition; differing regarding the TMR cueing protocol applied) (see Supplementary Fig. 4, displaying the expected change trajectories for negative valence).

## Results

Participant characteristics (*n* = 80) and emotional memory features are displayed in Table 1 (and Supplementary Table 11). At baseline, memory characteristics were afflicted with moderate to severe distress (see Supplementary Fig. 2 and Table 1). Heart rate (HR) was higher when confronted with the emotional (*M* = 76.35, SD = 11.57) compared to a neutral memory script (*M* = 72.39, SD = 11) (*t* = 1.9033, df = 115.7, *p* = 0.03) (see Supplementary Fig. 3). The four study groups did not differ significantly in any characteristic at baseline (see Supplementary Tables 1 and 2). A CONSORT participant flow diagram is provided in the Supplementary Fig. 1.

### ImR effects on emotional memory characteristics

ImR was associated with significant changes in all memory characteristics from pre- to post- ImR (t1 vs. t2; see Supplementary Fig. 2 and Table 1). Specifically, negative valence (*d* = 1.64; *t*(79) = 14.7, *p* < 0.001), emotional distress (*d* = 1.66; *t*(79) = 14.89, *p* < 0.001), arousal (*d* = 1.23; *t*(79) = 10.99, *p* < 0.001) and vividness (*d* = 0.76; *t*(79) = 6.8, *p* < 0.001) decreased, and positive valence increased (*d* = 0.86; *t*(79) = −7.67, *p* < 0.001). Following ImR, participants also reported significantly less distressing memory-related negative beliefs (*d* = 0.85; *t*(79) = 7.48, *p* < 0.001).

### Sleep parameters and TMR effects on emotional memory characteristics

Total sleep time (TST) and number of words played identified by the automatic sleep stage scoring algorithm are displayed in the Supplementary Table 3. On average, 248 words (SD = 128; Range 15–596) were played per night and 92% of these cues were played during NREM sleep (N2 sleep 78.34%; N3/SWS sleep 13.92%) (see Supplementary Table 4). The study groups did not differ significantly in any of these sleep indices (see Supplementary Table 3). There were no differences in physiological sleep architecture between study groups (TMR condition) when the cues were presented or not (see Supplementary Table 5). At the debriefing, 32% of participants reported that they had heard words during sleep (total *n* = 19; EG-1: *n* = 8; EG-2: *n* = 5; CG-2: *n* = 6; no significant group differences were found (*χ*^2^ (2, *N* = 80) = 0.08, *p* = 0.96)) and word hearing was included as a control variable. To demonstrate effectiveness of cue presentation, we compared cue-associated event-related potentials (ERPs) and found that the cued groups (EG and CG-2) exhibited elevated cue-related ERPs during the first cueing night, while CG-1, which had no cues, did not show increased ERPs (see Supplementary Fig. 9).

**Fig. 2:**
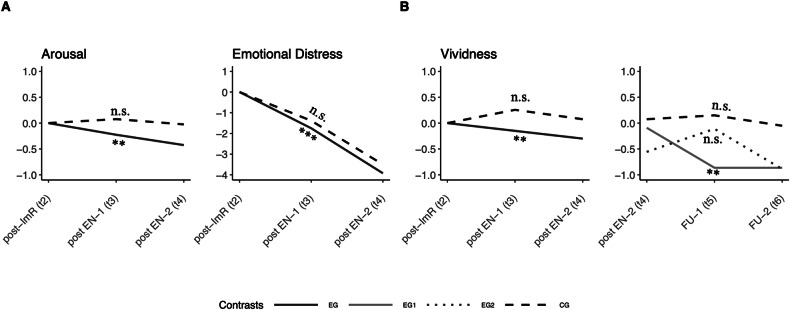
Change score trajectories centered at post-ImR (t2) for selected emotional memory characteristics. Visualization of change score trajectories after post-ImR (t2) for the four contrasts according to the preregistered hypotheses. Memory characteristics were centered to their corresponding post-ImR score (tx–t2). **A** From post-ImR (t2) to post-experimental nights (EN; t3–t4) change score trajectories significantly decreased in arousal and emotional distress in the EG receiving ImR cueing, whereas changes in the CG were not significant. **B** From post-ImR (t2) to post-experimental nights (t3–t4) change score trajectories significantly decreased for vividness in the EG receiving ImR Cueing, whereas changes in the CG were not significant (left panel). From the first two experimental nights (t4) to the follow-up (FU) appointments (t5–t6) further significant decrease in change score trajectories occurred in vividness only in EG-1 receiving three additional ImR Cueing nights, whereas further changes in EG-2 receiving three additional EEG-sham nights without cueing where not significant (right panel).

Results of the multilevel analyzes and the preregistered change trajectories in memory characteristics across assessments (time points, *t*) between study groups are summarized in Table 2 and Fig. 2. We found that the above reported within-session ImR-changes were maintained across timepoints, as indicated by significant ImR main effects on all emotional memory characteristics across groups (TMR condition, see Table 2 contrast “Trend overall ImR”). In addition to these ImR main effects, the EG, receiving cueing with words from the ImR updated memory, showed significant additional changes in memory characteristics, as indicated by significant decreases in arousal (*b* = −0.03; *p* = 0.006), emotional distress (*b* = −0.03; *p* < 0.001), and vividness (*b* = −0.09; *p* = 0.008) in the EG only (contrast “Trend EG”), while no further significant changes occurred in the CG (contrast “Trend CG”). We found no evidence of any cueing effects on positive and negative valence (see Table 2) nor on negative belief distress (see Supplementary Table 6). We observed a dose-response relationship for vividness, indicating further significant decrease in vividness in the EG-1 (three additional cueing nights, contrast “Trend EG-1”: *b* = −0.26, *p* = 0.003) after t4 (vs. t5–t6), compared to no further significant changes in EG-2 (additional sham nights, contrast “Trend EG-2”). No TMR effects on HR were found (see Supplementary Fig. 3).

**Table 2:** Emotional memory characteristic trajectories: multivariate analysis contrasts and multilevel regression weights.

A					B				
Contrast	Time-points	TMR condition (study group)	Model expectation	Interpretation					
					Primary outcomes				
					Negative valence (1–10)	Positive valence*^1^ (1–10)	Arousal (1–10)	Vividness (1–10)	Emotional distress (11–110)
**Trend overall ImR**	t1 vs. t2–t6	same across conditions	sign.	Expected ImR main effect across the TMR conditions by comparing t1 pre-ImR against all other following time points (t2–t6)	−0.49 ***	−0.04***	−0.12***	−0.25***	−0.12***
**Trend CG vs. EG**	across 6 time-points	CG vs. EG	not sign.	Overall condition effects across timepoints	0.01	0.03	0.002	−0.18	0.06
**Trend CG**	t2 vs. t3–t6	CG only	not sign.	Expected no further change in memory characteristics after the experimental nights (from t3) in the CG, receiving no (CG-1) or cueing with neutral words (CG-2)	0.03	0.04***	0.004	0.02	−0.01
**Trend EG**	t2 vs. t3–t6	EG only	sign.	Expected further improvements in memory characteristics after the experimental nights (from t3) in the EG receiving ImR cueing	0.00	0.03***	−0.03**	−0.09**	−0.03***
**Trend CG-1 vs. CG-2**	across 6 time-points	CG-1 vs. CG-2	not sign.	Overall condition effect across timepoints	−0.08	−0.02	0.01	0.18	0.03
**Trend EG-1 vs. EG-2**	across 6 time-points	EG-1 vs. EG-2	not sign.	Overall condition effect across timepoints	0.02	−0.01	−0.01	−0.03	0.03
**Trend EG-1**	t4 vs. t5–t6	EG-1 only	sign.	Expected significant improvement in memory characteristics after t4 in EG-1 receiving three additional ImR cueing nights (difference between t4 compared to t5 and t6)	−0.14	0.02	−0.04	−0.26 **	−0.02
Trend EG-2*^2^	t4 vs. t5–t6	EG-2 only	not sign.	Expected no significant changes after t4 in EG-2 receiving no additional ImR cueing nights (but sham-EEG; no difference between t4 compared to t5 and t6)	−0.06	−0.02	−0.02	0.02	0.02
**CV heard cues**	across 6 time-points	EG and CG	no formulated hypothesis for the CV	Effect of the CV hearing the ImR related words	−0.21	0.19	−0.02	−0.43	−0.11
**CV heard cues:EG**	across 6 time-points	EG vs. CG		Effect of the CV hearing the ImR related words in the EG vs. CG (interaction)	1.31	−0.08	0.27	0.69	0.43

**A**. Multivariate analysis comprising eight contrasts to test the pre-registered hypothesized change trajectories in emotional memory characteristics across assessments (timepoints, *t*) between the study groups (TMR condition). Formulated significant model expectations regarding change trajectories of memory characteristics between study groups (TMR condition) and timepoints are based on the hypotheses. For a visualization of the defined contrasts, see Supplementary Fig. 4. **B**. Multilevel regression weights for the defined contrasts and the control variable (CV) whether participants heard the cues. *** *p* < 0.001, ** *p* < 0.01, * *p* < 0.05. In line with our pre-registered hypothesis, one-sided p-values are reported. *^1^Please note that due to the Box-Cox transformation of the outcome variable positive valence, a negative regression weight means an increase in positive valence and vice versa, see Supplementary Table 1 and Fig. 2. We found no cueing effects on positive valence, as the increase in positive valence within the ImR session (t1 vs. t2) decreased significantly again over time after the Experimental nights (t2 vs. t3–t6) in both the EG (contrast “Trend_EG”) and the CG (contrast “Trend_CG”). *^2^We repeated the analyzes without the five participants in EG-2 who refused the three additional EEG nights (no sham nights), which did not change the pattern of significant results (see Supplementary Table 8).

The control variable of whether participants heard the cues showed no significant effects (see Table 2), and removing this control variable from the analyzes did not change the pattern of significant results (see Supplementary Table 7). Additionally, we controlled for possible between-experimenter effects, which were not significant, and adding the control variable of the experimenters did not change the pattern of significant results (see Supplementary Table 9). Exploratively, we found no significant associations between the number of cues played on short (t3 and t4) and long-term (t5 and t6) TMR effects, all p’s > *p* = 0.05 (see Supplementary Table 10).

## Discussion

We report effects of ambulatory TMR applied during NREM sleep SWs following ImR on key emotional memory characteristics. A single-session ImR procedure was effective in modulating socially aversive memories, as indicated by large effect sizes in improvements in emotional and cognitive memory characteristics from pre- to post- ImR. With regards to the effects of TMR, our study identified additional changes in emotional memory characteristics following ImR in participants cued with ImR-related words compared to no or neutral word cueing during sleep. These additional improvements emerged in memory-related vividness, arousal, and emotional distress. Moreover, we presented evidence for a relationship between cueing dose and possible TMR- associated memory changes, namely, additional decreases in vividness in the group receiving five nights of ImR cueing compared to two. Changes in memory characteristics were maintained at one-week and one-month follow-up. Together, these results suggest that TMR may augment ImR, possibly by reactivating a ImR-modulated memory and enhancing its offline reconsolidation during sleep.

Our findings align with observations of previous studies showing that TMR applied to standardized emotional stimuli modulates emotional memory consolidation [21, 31]. We extended these findings and showed that using idiosyncratic words from an updated ImR-treated autobiographical memory were associated with changes in memory characteristics. TMR effects were, however, small. One explanation for this could be that TMR effects were additive to the effects of naturally occurring offline memory consolidation processes. After the evening ImR session, all participants slept at home, hence rendering effects of the control groups receiving no cueing or TMR with neutral words comparatively strong. It is also possible that memory “tagging” occurred naturally, as our participants may have perceived the ImR session and its respective memory modulation as relevant [64, 65]. In addition to emotional context, motivational factors may contribute to such a tagging effect that presumably increases the probability of access to offline reactivation, as emotions associated with a memory enhance naturally occurring offline memory consolidation processes [14, 15, 66].

Although the translational potential of TMR for clinical applications to augment psychotherapy treatments has been repeatedly addressed [35–37], the application of TMR to treat idiosyncratic autobiographical emotional memories has not yet been systematically investigated. In two notable exceptions, Rihm et al. [38] and Borghese et al. [67] administered TMR after reconsolidation of a fear memory adapted through exposure therapy in phobia patients. However, both found no significant TMR effects in addition to the effects of exposure therapy. In the study by Rihm et al. [38] this was partly explained by ceiling effects of the exposure procedure. In addition, treatment-realted olfactory cues were replayed once during a brief daytime nap in the laboratory, which might have been too brief to enhance extinction memory consolidation. Memory consolidation might involve dynamic and slow processes evolving over several nights of sleep [68, 69]. Borghese et al. [67] found no main effect of one week of TMR applied at home. They re-presented treatment-related acoustic cues during REM sleep. However, especially SWs occurring during NREM sleep have been associated with memory consolidation processes [13]. These procedures thus diverge from our design, where reminder cues from an updated memory were presented during NREM sleep following ImR. Regarding the time frame during which relevant consolidation processes unfold, cueing dose results could reveal further insights. Specifically, our results showed an additional reduction of vividness in the group receiving five compared to two cueing nights. In the present study, we can rule out expectation effects, as no such effects were observed in EG-2 receiving three additional sham nights without cueing. Vividness is a key characteristic of negative emotional memories, and in PTSD vivid and intrusive memories have been associated with a lack of adequate contextualization within autobiographical memory bases [70]. TMR may enhance the integration of the updated memory within pre-existing autobiographical memories and their contextualization, resulting in decreased vividness. Yet, our data cannot provide direct evidence for the neurophysiological mechanisms underlying the hypothesized TMR effects on ImR outcomes. The observed pattern of behavioral results and group differences, however, support the notion that TMR may bias offline reactivation and reconsolidation processes toward integration and stabilization of the adaptively modified memory trace. Future studies should find ways to incorporate measures that distinguish between the original and updated memory, e.g., to more directly measure memory updating and reconsolidation processes. This is currently challenging, e.g., due to the lack of direct neurobiological as well as behavioral measures of such memory processes, especially for complex and adverse autobiographical memories, but may be possible in the future [71–73].

With regard to ongoing oscillatory processes, in the current study, reactivation cues were presented in a non-phase-locked fashion via the MHSL-SB when the EEG signal reached certain delta thresholds indicating SW-rich NREM sleep [1, 46]. Most of the cues were replayed during SWs occurring during N2 followed by N3/slow wave sleep (SWS) sleep. Future studies should investigate whether adjusting the algorithm used in this study, e.g., setting a higher delta threshold and thus presenting cues only during deeper sleep, e.g., only during N3/SWS sleep, affects the pattern of results. In addition, recent work proposed that timing cues to depolarizing SWs up-states might promote TMR effects [36, 74]. However, the evidence on the optimal stimulus phase is inconclusive [75–78], and studies examining the optimal timing of cueing in the context of emotional memory consolidation are lacking. Our results of positive TMR effects during SWs rich NREM sleep are promising and align with empirical findings, that N2 sleep, N3/SWS and sleep spindles play a key role in offline consolidation and integration of new information into existing memory structures [79, 80]. Furthermore, a recent meta-analysis suggested that across memory domains (e.g., declarative, procedural and emotional memories) TMR is most effective when cues were represented during N2 and N3/SWS sleep [20]. According to the sequential processing hypothesis [81], memory consolidation might also benefit from the cyclic sequence of NREM and REM sleep, such that REM sleep might further promote and strengthen the integration of new memories previously re-activated and consolidated within long-term memory networks during NREM sleep SWs [82–84]. In addition, re-processing of emotional memory content during REM sleep may reduce its affective tone reflected in lower memory distress ratings [85, 86]. However, future studies should investigate whether the precise timing of cues regarding ongoing oscillatory processes could further improve TMR effects.

In addition, we found positive effects of TMR for some, but not all, memory characteristics, namely vividness, arousal and emotional distress, and no effects on positive and negative valence. Further research is needed to determine which characteristics and symptoms of aversive memories are amenable to TMR improvements.

The current study is not without limitations. Our sample consisted of young and healthy sleepers who reported distressing socially aversive memories. However, the selected memories (e.g., bullying experiences, rejection in a relationship) had characteristics similar to those of distressing memories in clinical samples, such as for example the presence of a perpetrator or heightened distress in memory-related characteristics such as emotional distress, arousal, vividness, and intrusions [87]. Translation to clinical samples such as people suffering from PTSD, anxiety and/or sleep disorders is thus warranted. Furthermore, we did not include a group receiving TMR with memory reactivation cues only. We can thus not exclude that TMR reactivation of a previous distressing memory (without ImR) could promote and reinstate normal consolidation processes, thereby improving afflicting negative emotional memory characteristics [88]. Future studies should thus include additional control conditions. Furthermore, although the majority of participants, 68%, did not hear the words, we did not succeed in presenting the cues on a subliminal level to all participants. However, hearing the cues did not affect the significant patterns in the results. Our results indicated that the presence of TMR cues had no effect on sleep architecture structures. However, we cannot exclude the possibility that TMR cues lead to changes in microstructures of sleep, such as arousals, etc., which should be investigated in future studies. Finally, future studies should investigate whether phase-locking of reminder cues to SWs up-phases or specifically targeting SWs during N3/SWS further enhances TMR’s effects on modulation of emotional memories.

Taken together, our results support TMR as a non-invasive method to reactivate specific memories during sleep following memory and learning interventions, in our study by using personalised ImR words stimuli. We found no evidence of negative effects of TMR on emotional memory characteristics or sleep phase architecture, which adds to the body of evidence supporting the translation of TMR research to clinical samples. However, clinical samples are often characterized by disturbed sleep conditions [89]. Future research will therefore need to investigate TMR under pathological sleep conditions, e.g., whether TMR could promote the likelihood of adaptive consolidation effects even under unfavourable sleep conditions and whether it may even have the potential to counteract negative effects of pathological sleep conditions [36, 37, 90]. Many current psychotherapy interventions, e.g., in CBT, rely on modulating memories associated with traumatic or stressful events, e.g., through memory updating or extinction learning processes. However, these methods are in dire need for improvement [40, 41] and the targeted reactivation of treatment memories during sleep could be a promising avenue offering tantalizing options for personalized treatment approaches. While most TMR studies, with some exceptions [91, 92], have been conducted on single nights in the sleep laboratory, we show that TMR applied through wearable EEG systems can be incorporated into daily routines in participant’s homes over multiple nights. Future studies should expand these results to clinical settings and investigate whether TMR could be used to optimise psychotherapy treatment targeting specific psychotherapy sessions or accompanying the course of CBT across several weeks to improve treatment outcomes.

## Supplementary information

Supplementary Material

## Data Availability

All processed data necessary to interpret, verify, and extend the main results of this study are available in the Source Data file on OSF https://osf.io/qmav9/.

## Code availability

Original code used for data analysis is available on OSF https://osf.io/qmav9/.
