# Supplementary material for: Targeted memory reactivation during sleep improves emotional memory modulation following imagery rescripting
Source: Transl Psychiatry. 2024 Dec 18;14:490. doi: 10.1038/s41398-024-03192-4 (PMC11655627; doi:10.1038/s41398-024-03192-4)
Supplement: Supplementary file 1 — Supplementary Material [file 41398_2024_3192_MOESM1_ESM.pdf]

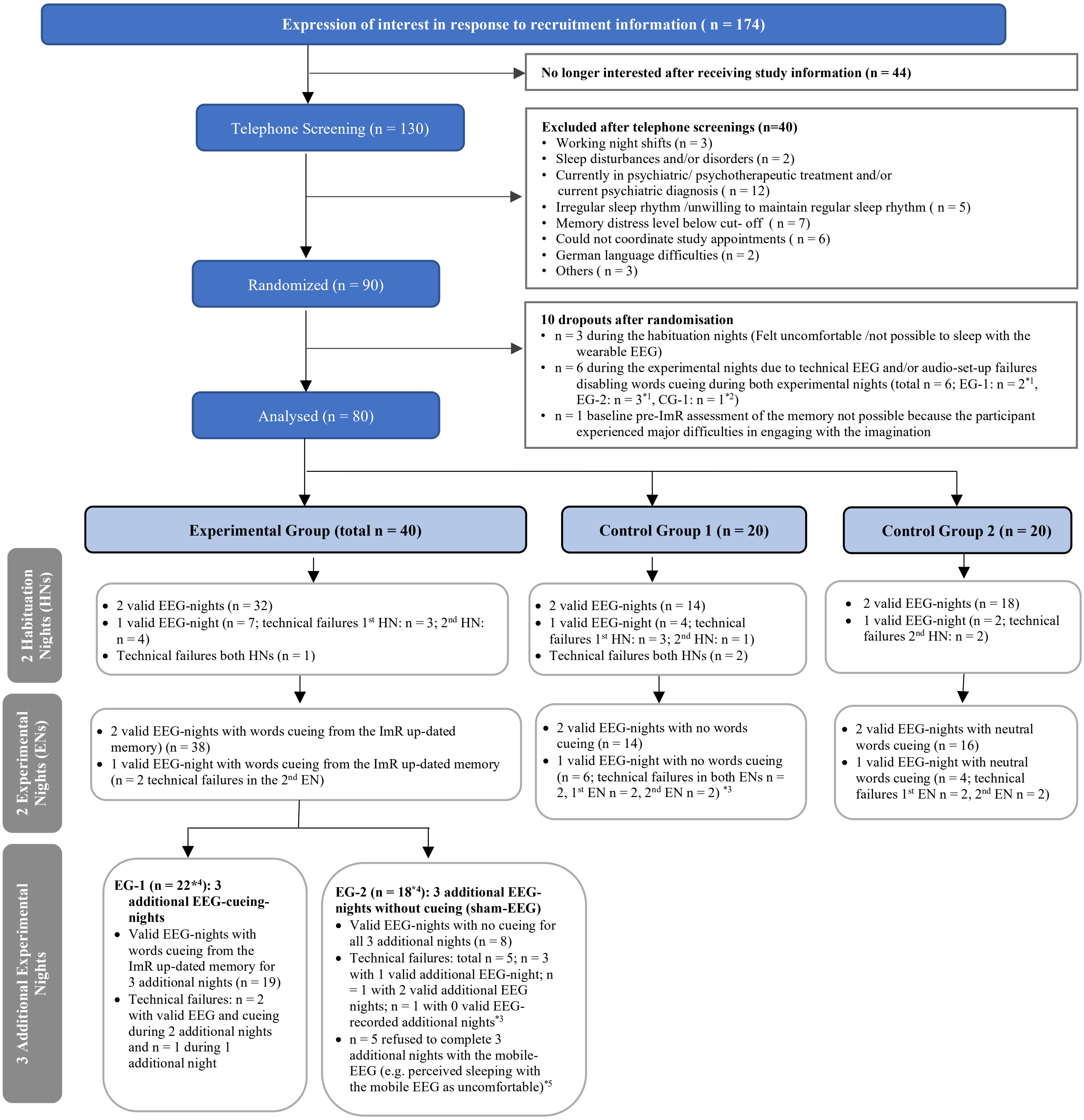


**Supplementary Figure 1| Participant flow diagram summarizing sampling procedures and available EEG-nights data with valid words cueing setups.**

Due to technical artifacts and failures of the wearable EEG (MHSL-SB), the number of valid EEG-night recordings with valid cueing setups varied. All participants with at least one valid EEG-night recording and valid cueing setup were retained in the final sample (n = 80). EEG = Electroencephalography, EN = experimental night, HN = habituation night.

[*1]Not possible to include these participants in the analysis, e.g. in CG-1 (receiving no words cueing), as they all completed 3 additional MHSL-SB nights and thus differ from CG-1.

[*2] One Participant had to be excluded because pink noise was played in deep sleep after a faulty device update possibly influencing Slow waves (SWs ) characteristics.

[*3] Since the participants did not subjectively perceive the invalid EEG recordings (the device could be switched on, but no valid EEG recording was possible due to technical failures) and valid EEG recordings were not relevant in both CG-1 (for the two experimental nights) and EG-2 (for the three additional experimental nights) - all characterized by nights without word cueing - these nights were considered valid control nights without cueing (sham condition) and thus included in the main analyses.

[*4] For n = 2 participants initially randomized to EG-2 the cueing could not be deactivated remotely due to participant's WLAN provider not allowing for remote access to the device. Therefore, the subjects had five cueing nights and are included in the analyses in EG-1, resulting in n = 22 in EG-1 and n = 18 in EG-2.

[*5] n = 5 participants (n = 3 randomized to EG-1 and n = 2 to EG-2) refused to complete the three additional experimental nights. In order to still utilize their data in the exploratory analyses of possible dose-response relationships of the cueing effects in the EG, they were re-assigned to EG-2 (n =3) or remained in EG-2 (n =2). We repeated the analyses without the five participants in EG-2 who refused the three additional EEG nights (no sham nights), which did not change the pattern of significant results (see Supplementary Table 8).

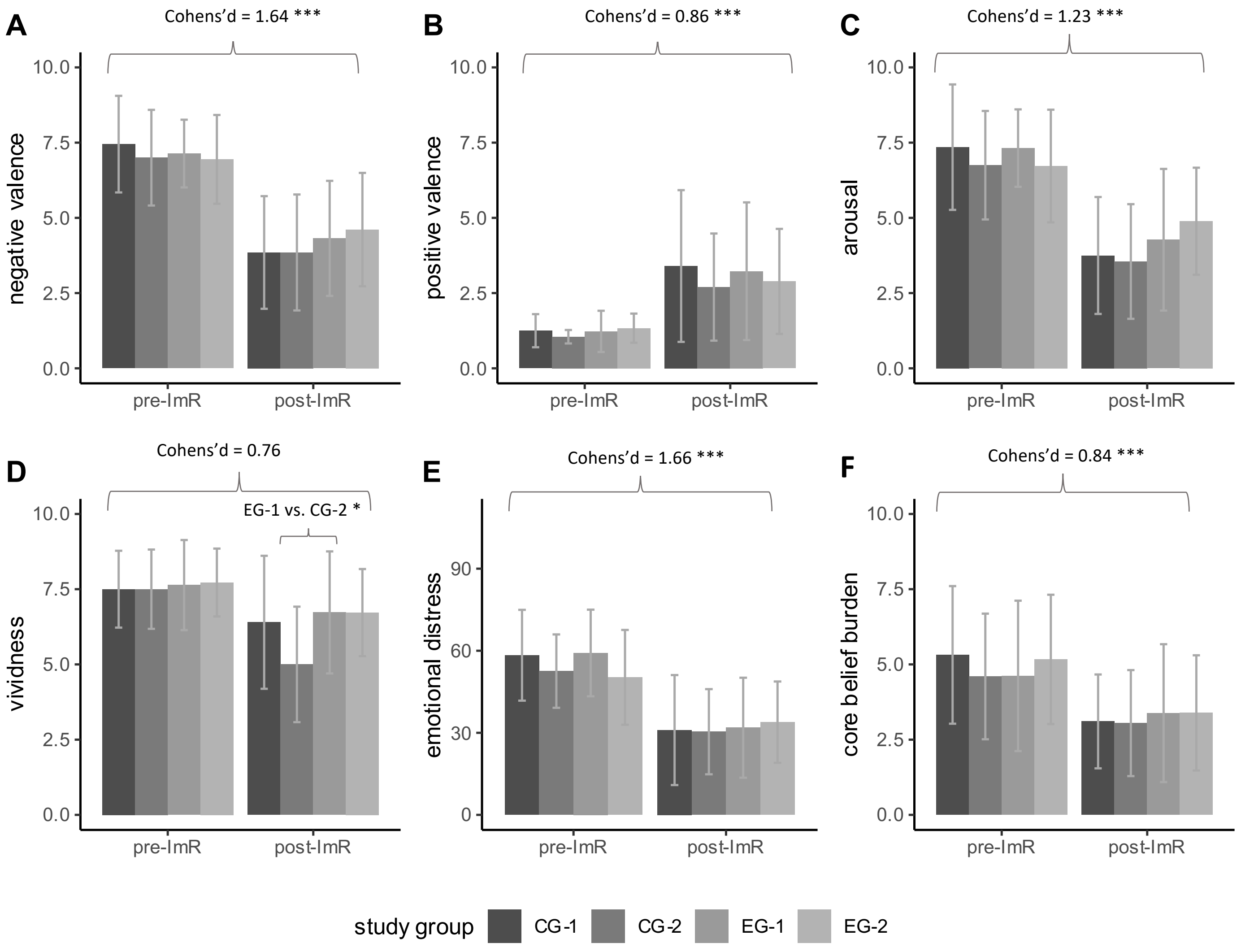


**Supplementary Figure 2 | Within ImR session changes in memory characteristics by study group.**

Changes in memory characteristics (primary outcomes) from pre- (t1) to post-(t2) ImR in negative and positive valence, vividness, arousal, and emotional distress, as well as distress associated with negative memory-related beliefs (secondary outcome). All memory characteristics were indexed on a 10-point Likert–type scale from 1 "not at all" to 10 "very strongly", except for emotional distress, which was assessed on a Likert-type scale form 11 – 110. All Cohen's d show significant within-ImR-session changes that fall in the medium to high effect size range. With the exception of vividness post-ImR, which was significantly higher in EG-1 compared to CG-2, there were no other statistically significant differences in memory characteristics between the groups pre- (t1) and post-(t2) ImR (see also Supplementary Table 1). However, to investigate the effects of cueing on the first two experimental nights (EN 1-2), both EGs were analysed together and compared with the two combined CGs.

## Supplementary Table 1 | Emotional memory characteristics at t1 and t2 by study group

| Time. | Total (n = 80) | EG-1 (n = 22) | EG-2 (n = 18) | CG-1 (n = 20) | CG-2 (n = 20) | F-statistics group-differences |
|---|---|---|---|---|---|---|
| **Negative valence** | | | | | | |
| **t1** | 7.14 (SD = 1.44; Range: 4 – 10) | 7.14 (SD = 1.13) | 6.94 (SD = 1.47) | 7.45 (SD = 1.61) | 7 (SD = 1.59) | F(3,76) = 0.47, p = 0.70 |
| **t2** | 4.15 (SD = 1.89; Range 1-8) | 4.32 (SD = 1.91) | 4.61 (SD = 1.88) | 3.85 (SD = 1.87) | 3.85 (SD = 1.93) | F(3,76) = 0.74, p = 0.53 |
| **Emotional distress (EIBE)** | | | | | | |
| **t1** | 55.31 (SD = 15.97; Range: 23 – 98) | 59.18 (SD = 15.83) | 50.28 (SD = 17.32) | 58.35 (SD = 16.6 ) | 52.55 (SD = 13.41) | F(3,76) = 1.5, p = 0.22 |
| **t2** | 31.74 (SD = 17.13; Range: 11 -86) | 31.86 (SD = 18.27) | 33.89 (SD = 14.87) | 31 (SD = 20.1) | 30.4 (SD = 15.57) | F(3, 76) = 0.14, p =0.93 |
| **Arousal** | | | | | | |
| **t1** | 7.05 (SD = 1.76; Range: 2 – 10) | 7.32 (SD = 1.29) | 6.72 (SD = 1.87) | 7.35 (SD = 2.08) | 6.75 (SD = 1.8) | F(3,76) = 0.76, p = 0.52 |
| **t2** | 4.1 (SD = 2.05; Range 1-9) | 4.27 (SD = 2.35) | 4.89 (SD = 1.78) | 3.75 (SD = 1.94) | 3.55 (SD = 1.9) | F(3,76) = 1.66, p = 0.18 |
| **Positive Valence** | | | | | | |
| **t1** | 1.21 (SD = 0.52; Range: 1-4) | 1.23 (SD = 0.69) | 1.33 (SD = 0.49) | 1.25 (SD = 0.55) | 1.05 (SD = 0.22) | F(3,76) = 1.02, p = 0.40 |
| **t2** | 3.06 (SD = 2.10; Range 1 -10) | 3.23 (SD = 2.29) | 2.89 (SD = 1.75) | 3.4 (SD = 2.52) | 2.7 (SD = 1.78) | F(3,76) = 0.45, p = 0.72 |
| **Vividness** | | | | | | |
| **t1** | 7.59 (SD = 1.30; Range: 5-10) | 7.64 (SD = 1.5) | 7.72 (SD = 1.13) | 7.5 (SD = 1.28) | 7.5 (SD = 1.32) | F(3,76) = 0.13, p = 0.94 |
| **t2** | 6.21 (SD = 2.03; Range 2-10) | 6.73 (SD = 2.03) | 6.72 (SD = 1.45) | 6.4 (SD = 2.21) | 5 (SD = 1.92) | F(3,76) = 3.61, p = 0.017 *[1] |
| **Distress negative belief** | | | | | | |
| **t1** | 4.88 (SD = 2.25; Range: 1-9) | 4.62 (SD = 2.5) | 5.17 (SD = 2.15) | 5.32 (SD = 2.29) | 4.47 (SD = 2.06) | F(3,73) = 0.63, p = 0.60 |
| **t2** | 3.23 (SD = 1.88; Range: 1-10) | 3.38 (SD = 2.29) | 3.39 (SD = 1.91) | 3.11 (SD = 1.56) | 3.05 (SD = 1.76) | F(3,74) = 0.17, p = 0.92 |
| **Heart Rate (HR bpm)** | | | | | | |
| **t1** | 76.35 (SD = 11.57) | 77.13 (SD = 10.76 ) | 72.37 (SD = 11.94 ) | 74.95 (SD = 7.07 ) | 82.18 (SD = 14.84) | F(3,55) = 1.82, p = 0.16 |
| **t2** | 72.54 (SD = 10.32) | 72.19 (SD = 7.99) | 73.38 (SD = 10.88) | 70.63 (SD = 10.74) | 74.15 (SD = 12.75) | F(3,55) = 0.29, p = 0.83 |
| **t5** | 77.15 (SD = 11.32) | 76.33 (SD = 9.18) | 75.54 (SD = 11.95) | 78.37 (SD = 10.87) | 79 (SD = 14.44) | F(3,55) = 0.29, p = 0.83 |
| **Change score (HR)** | | | | | | |
| **t1** | 3.96 (SD = 7.4) | 2.15 (SD = 4.85) | 2.82 (SD = 4.46) | 3.23 (SD = 6.5) | 8.88 (SD = 11.97) | (F3,55) = 2.46, p = 0.07 |
| **t2** | 1.4 (SD = 8.95) | -2.13 (SD = 7.09) | 5.86 (SD = 8.93) | -1.41 (SD = 9.75) | 3.74 (SD = 7.96) | F(3,55) = 0.29, p = 0.83 |
| **t5** | 0.71 (SD = 6.1) | -0.86 (SD = 4.65) | 1.73 (SD = 3.74) | 2.34 (SD = 6.58) | -0.34 (SD = 9.19) | F(3, 55) = 0.29, p = 0.83 |

There were no significant differences in memory characteristics between the four study groups at t1 (baseline, pre-ImR). At t2 (post-ImR), pairwise comparisons using t tests with pooled SD showed that there was a significant difference between CG-2 and EG-1 only for vividness (p = 0.017).

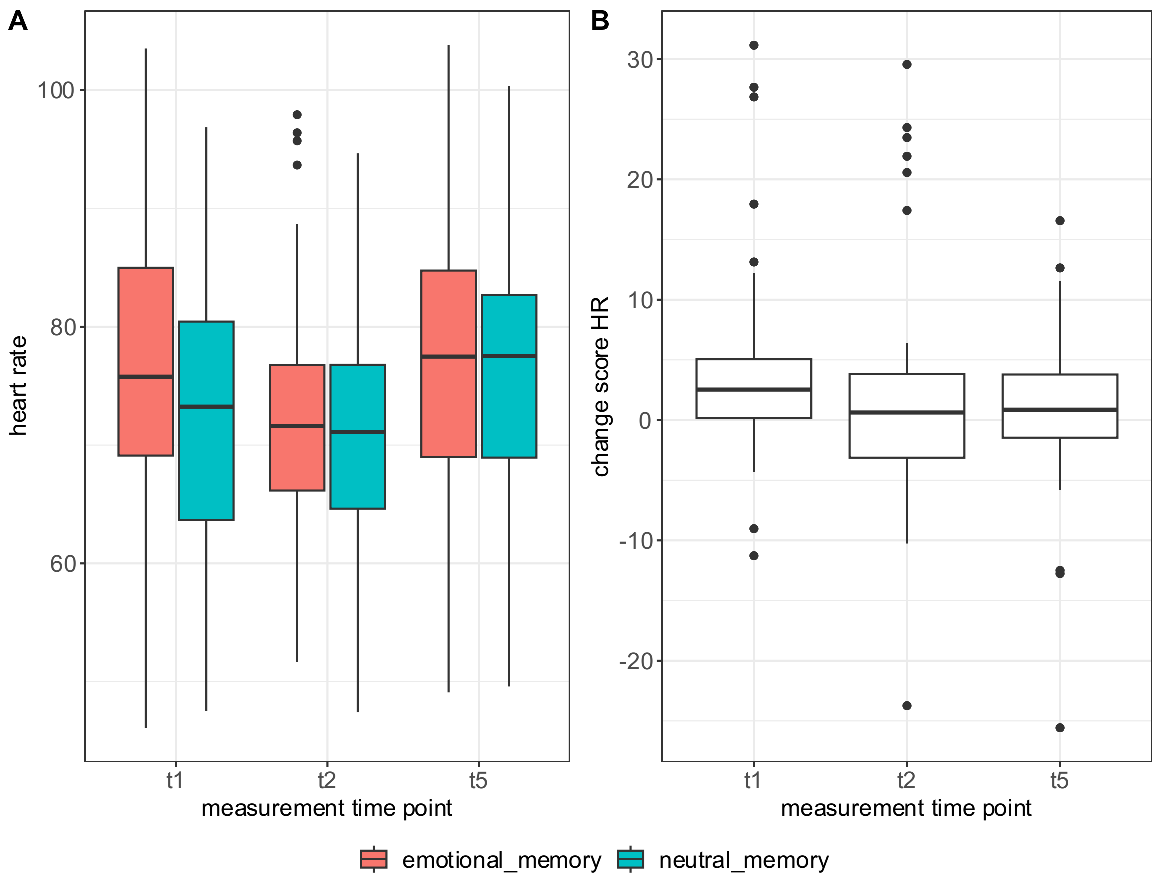


**Supplementary Figure 3 | Heart rate (HR) activity (beats per minute, bpm) and HR change scores.**

**A.** Boxplots depicting mean HR and standard deviations at pre- (t1) and post-ImR (t2) and 1-week follow-up (FU-1, t5). There was only a marginally significant effect pointing in the assumed direction of a reduced HR post- (t2) compared to pre-ImR (t1) ($d = 0.21$; $t(58) = 1.59$, $p = 0.059$). **B.** Boxplots depicting HR change scores (HR imaging emotional - HR imaging neutral memory script; a positive value indicates a higher HR when imaging the emotional memory script) at three different time points pre- (t1, evening) and post- ImR (t2, evening), and 1-week FU (t5, morning). We found no evidence of significant TMR effects on HR at 1-week follow-up (t5; $t(36) = 0.05$, $p = 0.48$).

**HR data preprocessing and description of the intervals of the script driven imagery procedure**. To calculate HR response to the emotional memory script for each of the 3 timepoints pre- ($t_1$) and post-ImR ($t_2$), and 1-week FU ($t_5$), participants were asked by the experimenter to set markers on the E4 wristband. For example, one marker was set at the beginning of the script driven imagination, followed by the subsequent intervals: I) the instruction to adopt a comfortable position, relax and introspect to the breath (60 seconds), II) the instruction to slowly wander back in time, the reading aloud of the memory script by the experimenter and the instruction to let the situation and the surroundings vividly arise before one' s inner eye (60 seconds), followed by III) a 30-second re-experiencing of the situation with as many senses as possible. In order to control for possible inter-individual differences in HR baseline across the 3 time points (e.g. due to circadian effects), HR change scores were calculated by subtracting the 90-s re-experiencing intervals (intervals II and III) of the neutral from the emotional memory scripts for each time point. HR data count was reduced due to technical artefacts and failures (n = 5 at t1 and t2 (EG: n = 3; CG-1: n = 1; CG-2: n = 1); N = 8 at t5 (EG-1: n = 3, CG-1: n = 2, CG-2: n = 3)). HR data of six participants exceeding 1.5 x the interquartile range of the HR values measured (indexed separately for the emotional and neutral scripts) sample distribution were further excluded from the analysis (1).

## Supplementary Table 2 | Descriptive and clinical sample characteristics by study group

| Variable | EG-1 (n = 22) | | EG-2 (n = 18) | | CG-1 (n = 20) | | CG-2 (n = 20) | | F-Statistic: sample characteristic x study group |
|---|---|---|---|---|---|---|---|---|---|
| | Mean | SD | Mean | SD | Mean | SD | Mean | SD | |
| Age | 23.77 | 5.04 | 21.83 | 2.81 | 23.45 | 2.89 | 22.6 | 3.23 | F(3, 76) = 1.11, p = 0.36, eta2[g] = 0.04 |
| Gender | n = 15 female, n = 7 male | | n= 13 female, n = 5 male | | n = 14 female, n = 6 male | | n = 16 female, n = 4 male | | $x^2$ (3, $N$ = 80) = 0.8, $p$ = 0.84 |
| BAI | 8.5 | 7.19 | 6.9 | 5.45 | 9.4 | 6.72 | 8.55 | 8.54 | F(3, 76) = 0.41, p = 0.75, eta2[g] = 0.02 |
| BDI | 7.05 | 7.59 | 6.61 | 4.45 | 7.65 | 5.99 | 6.8 | 5.42 | F(3, 76) = 0.11, p = 0.96, eta2[g] = 0.004 |
| PSQI | 4.16 | 1.98 | 4.39 | 1.72 | 3.8 | 1.7 | 4 | 1.68 | F(3, 71) = 0.37, p = 0.77, eta2[g] = 0.02 |
| Age at the time of the event | 16.05 | 7.02 | 14.89 | 4.64 | 16.82 | 5.99 | 16.15 | 6.53 | F(3, 76) = 0.32, p = 0.81, eta2[g] = 0.01 |
| Years since memory | 7.73 | 7.11 | 6.94 | 4.63 | 6.62 | 4.89 | 6.45 | 5.87 | F(3, 76) = 0.2, p = 0.80, eta2[g] = 0.01 |
| *[1]Extent to which memory influences self-perception | 6.27 | 2.12 | 6.5 | 2.07 | 6.55 | 2.14 | 6.2 | 2.12 | F(3, 76) = 0.13, p = 0.94, eta2[g] = 0.01 |

No significant group differences in the four study groups with regard to the descriptive and clinical sample characteristics. M = Mean; Mdn = Median; BAI = Beck Anxiety Inventory ((2,3); BDI = Beck Depression Inventory II (4); German version by (5); PSQI = Pittsburgh Sleep Questionnaire (6).

## Supplementary Table 3 | Sleep characteristics and cueing protocol: TST and number of cues played by study group

| | Total Sample (n = 80) | | Sub-groups Analyses | | | | | | | | | | | | | | |
|---|---|---|---|---|---|---|---|---|---|---|---|---|---|---|---|---|
| | | | **EG-1 (total n = 22)** | | | **EG-2 (total n = 18)** | | | **CG-1 (total n = 20)** | | | **CG-2 (total n = 20)** | | | **F-Statistics** | |
| | **TST (M, SD)** | **n[1]** | **TST (M, SD)** | **Cues (M, SD, range)** | **n[1]** | **TST (M, SD)** | **Cues (M, SD, range)** | **n[1]** | **TST (M, SD)** | **Cues (M, SD, range)** | **n[1]** | **TST (M, SD)** | **Cues (M, SD, range)** | **n[1]** | **group x TST** | **group x n cues** |
| **HN-1** | 7.14 (SD = 0.98) | 71 | 6.88 (SD = 0.96) | **x** | **21** | 7.29 (SD = 0.86) | **x** | **15** | 7.22 (SD = 0.79) | **x** | **15** | 7.22 (SD = 1.22) | **x** | **20** | F(3, 67) = 0.68, p = 0.57, eta2[g] = 0.03 | x |
| **HN-2** | 7.24 (SD = 1.14) | 70 | 7.25 (SD = 0.84) | **x** | **20** | 7.20 (SD = 1.13) | **x** | **15** | 7.26 (SD = 1.17) | **x** | **17** | 7.23 (SD = 1.46) | **x** | **18** | F(3,66) = 0.007, p = 0.1, eta2[g] = < 0.00 | x |
| **EN-1** | 7.02 (SD = 1.11) | 75 | 6.92 (SD = 0.8) | 278 (SD = 136; 98 – 563) | **22** | 6.93 (SD = 1.32) | 237 (SD = 107; 60 – 424) | **18** | 6.87 (SD = 1.31) | **x** | **16** | 7.35 (SD = 1.03) | 238 (SD = 120; 56 – 561) | **18** | F(3,71) = 0.77, p = 0.52, eta2[g] = 0.03 | F(2,55)) = 0.76, p = 0.47, eta2[g] = 0.03 |
| **EN-2** | 6.87 (SD = 1.27) | 73 | 6.92 (SD = 0.9) | 286 (SD = 124; 69 – 585) | **21** | 6.87 (SD = 0.91) | 190 (SD = 101; 52 – 435) | **17** | 7.26 (SD = 0.77) | **x** | **16** | 6.46 (SD = 2.04) | 203 (SD = 135; 16 – 448) | **18** | F(3, 69) = 1.14, p = 0.34, eta2[g] = 0.05 | F(2, 53) = 3.597, **p = 0.03***, eta2[g] = 0.12;; sign. difference btw. EG1 and EG2[2] |
| **EN-3 (only EG)** | 6.95 (SD = 1.61) | 31 | 7.25 (SD = 0.73) | 260 (SD = 158; 15 – 596) | **21** | 6.33 (SD = 2.61) | **x** | **10** | **x** | **x** | **x** | **x** | **x** | **x** | F(1, 29) = 2.31, p = 0.14; eta2[g] = 0.07 | x |
| **EN-4 (only EG)** | 7.10 (SD = 0.90) | 29 | 7.17 (SD = 0.92) | 243 (SD = 137; 41 – 482) | **21** | 6.92 (SD = 0.87) | **x** | **8** | **x** | **x** | **x** | **x** | **x** | **x** | F(1,27) = 0.44, p = 0.51; eta2[g] = 0.02 | x |
| **EN-5 (only EG)** | 6.74 h (SD = 0.96) | 32 | 6.7 (SD = 1.1) | 267 (SD = 116; 18 – 481) | **21** | 6.79 (SD = 0.68) | **x** | **11** | **x** | **x** | **x** | **x** | **x** | **x** | F(1, 30) = 0.06, p = 0.81; eta2[g] = 0.002 | x |

M = Mean; SD = standard deviation; HN 1 -2 = habituation-nights 1 – 2; EN 1 – 5 = experimental-nights 1 – 5; x = no cueing. TST (in hours) was determined using a deep learning based automatic sleep stage scoring algorithm. [1] Due to some technical EEG recording errors, the number of valid EEG recordings and nights with a valid cueing setup varied by study night and group (see Supplementary Figure 1).

ANOVAs were applied to calculate the statistical differences between the four subgroups of the study. [2]Tukey post-hoc analyses revealed that only in EN-2 the EG-1 received significantly more cues than the EG-2 (-59.8, 95% CI (-191 to -0.17), adjusted p = 0.0495), but both groups were analysed together for cueing effects of the first two experimental nights (EN 1-2).

## Supplementary Table 4 | Total number of words played per sleep phase (in %)

| Sleep Phase | % of total words played |
|---|---|
| Wake | 1.11 |
| N1-Sleep | 1.10 |
| N2-Sleep | 78.34 |
| N3-Sleep | 13.92 |
| REM | 6.23 |

Percentage of total words played per sleep phase. Sleep stages were determined using a deep learning based automatic sleep stage scoring algorithm. N = nonREM sleep phases 1 -3; REM = rapid eye movement sleep.

## Supplementary Table 5 | Sleep duration per sleep phases by study group (TMR condition)

| | sleep duration per study phase EN1 | | | | | | | | | sleep duration per study phase EN2 | | | | | | | | |
|---|---|---|---|---|---|---|---|---|---|---|---|---|---|---|---|---|---|---|
| | EG-1 | | EG-2 | | CG-1 | | CG-2 | | F-statistics: group x sleep phase | EG-1 | | EG-2 | | CG-1 | | CG-2 | | F-statistics: group x sleep phase |
| | M | SD | M | SD | M | SD | M | SD | | M | SD | M | SD | M | SD | M | SD | |
| Wake | 0.59 | 0.39 | 0.53 | 0.4 | 0.63 | 0.79 | 0.57 | 0.37 | F(3, 70) = 0.12, p = 0.95, eta2[g] = 0.01 | 0.64 | 0.52 | 0.57 | 0.46 | 0.63 | 0.45 | 1.31 | 1.56 | F(3, 68) = 2.81, p = 0.046*[1], eta2[g] = 0.11 |
| N1 | 0.51 | 0.3 | 0.47 | 0.3 | 0.43 | 0.2 | 0.54 | 0.29 | F(3, 70) = 0.50, p = 0.68, eta2[g] = 0.02 | 0.48 | 0.23 | 0.44 | 0.27 | 0.46 | 0.24 | 0.65 | 0.49 | F(3, 68) = 1.48, p = 0.23, eta2[g] = 0.06 |
| N2 | 3.75 | 0.88 | 3.56 | 1.4 | 3.5 | 1.03 | 3.83 | 1.23 | F(3, 70) = 0.34, p = 0.80 eta2[g] = 0.01 | 3.83 | 1.18 | 3.3 | 1.02 | 4.12 | 1.39 | 3.98 | 1.3 | F(3, 67) = 1.43, p = 0.24 eta2[g] = 0.06 |
| N3 | 1.04 | 0.5 | 1.18 | 0.49 | 1.13 | 0.48 | 1.02 | 0.49 | F(3, 70) = 0.44, p = 0.73 eta2[g] = 0.02 | 1.12 | 0.46 | 1.29 | 0.38 | 1.13 | 0.47 | 0.88 | 0.42 | F(3, 67) = 2.56, p = 0.06 eta2[g] = 0.10 |
| REM | 1.61 | 0.74 | 1.72 | 0.76 | 1.8 | 0.87 | 2.11 | 0.97 | F(3, 69) = 1.25, p = 0.30 eta2[g] = 0.05 | 1.61 | 0.85 | 1.94 | 0.61 | 1.55 | 0.72 | 1.3 | 0.72 | F(3, 65) = 2.12, p = 0.11 eta2[g] = 0.09 |

Sleep duration (in hours) per sleep phase for the two experimental nights (EN-1; EN-2). There were no significant differences in sleep duration of different sleep phases between the study groups (TMR conditions; with cueing: EG-1, EG-2, CG-2 vs. without cueing: CG-1) in either EN-1 nor EN-2. Sleep phases were determined using a deep learning based automatic sleep stage scoring algorithm. [1] While the results of the general ANOVA indicated a significant effect of the study groups, the post-hoc Tukey HSD test showed no significant differences between the 4 study groups.

## Supplementary Table 6 | Change trajectories in distress associated with negative beliefs (secondary outcome): Multilevel Regression Weights

| Contrast | Distress negative belief (1 - 10) |
|---|---|
| **overall_ImR _trend** | - 0.07 (p < 0.001)*** |
| **CG_EG** | - 0.01 |
| **trend_CG** | 0.01 |
| **trend_EG** | 0.01 |
| **CG1_CG2** | 0.07 |
| **EG1_EG2** | - 0.04 |
| **trend_EG1** | 0.02 |
| **trend_EG2** | 0.04 |
| **CV heard cues** | 0.24 |
| **CV heard cues:EG** | 0.01 |

Notes: Multilevel Beta Regression Weights for the 8 eight hypothesis-based contrasts and the control variable (CV) whether participants heard something or not. *** $p < 0.001$, ** $p < 0.01$, * $p < 0.05$.

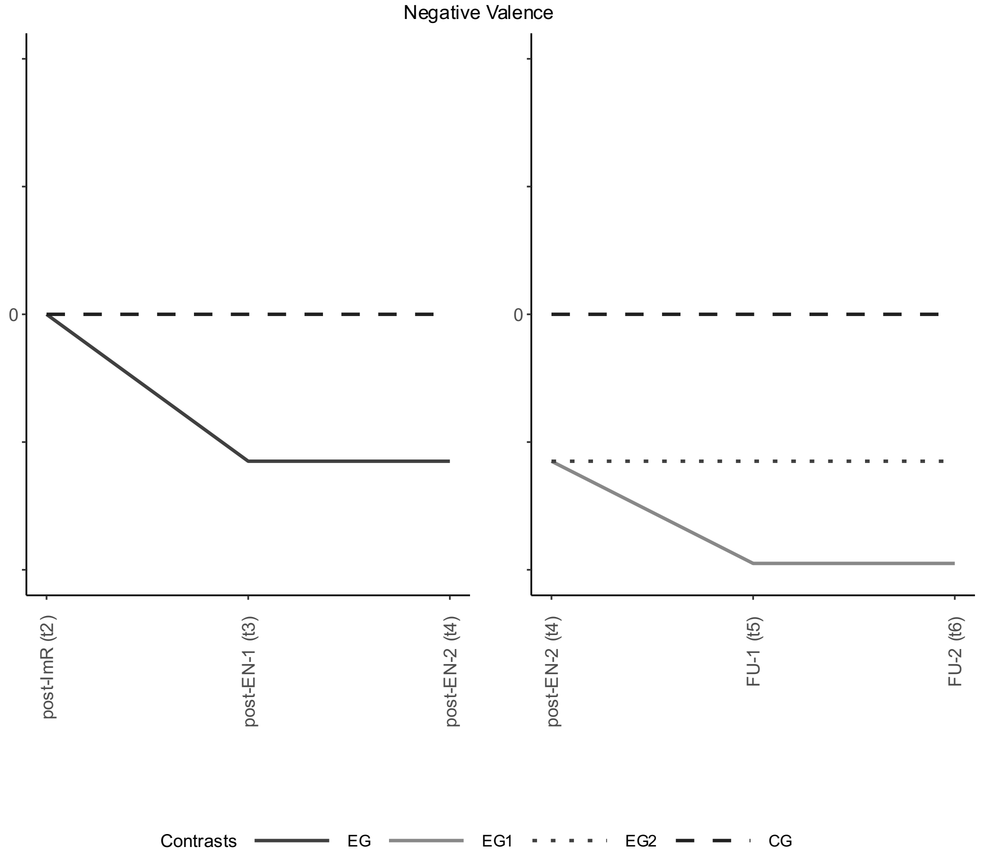


**Supplementary Figure 4 | Hypothetical change trajectories for the example of negative valence.**

Illustration of the hypothesized change score trajectories in the experimental and control groups of the multivariate model using simulated data and the example of negative valence. Depicted are the courses of the four contrasts for which we expected specific change trajectories according to our preregistered hypothesis. Namely, further significant improvements in the trends of the EG, EG-1, and EG-2 receiving TMR after t2, and no further significant changes in the CG receiving no or TMR with neutral words after t2 (left panel) (see Table 2 Main Study). In addition, we expected a further significant improvement in negative valence in EG1, receiving three additional nights of TMR cueing after t4, compared to no further significant changes in EG2, receiving three additional sham nights, and the CG receiving no TMR (right panel) (see Table 2 Main Study). To identify further change trajectories post-ImR (t2) in emotional memory characteristics across time points (t3 - t6) between study groups (TMR condition), emotional memory characteristics were centered at their corresponding post-ImR score (tx - t2).

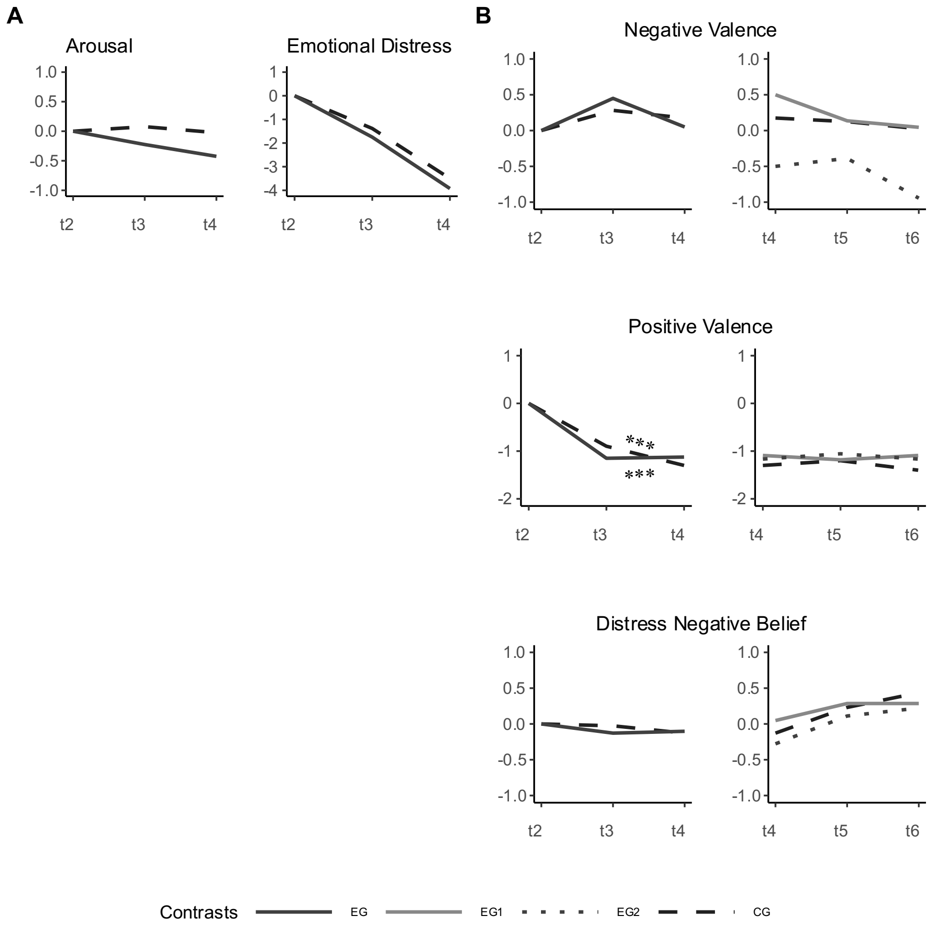


**Supplementary Figure 5 | Standardized change trajectories in selected emotional memory characteristics.**

Visualization of change scores post-ImR (t2). Depicted are the courses of the 4 contrasts for which we expected specific change trajectories according to our preregistered hypothesis, namely, further significant improvements in the Trend EG, EG-1, and EG-2 contrasts and no further significant changes in CG (see Table 2 Main Study). To identify further change trajectories post-ImR in emotional memory characteristics across time points (t3 - t6) between study groups (TMR conditions), emotional memory characteristics were standardized to their corresponding post-ImR score (tx - t2).

A. Graphical illustration of contrast Trend EG1, Trend EG2 and Trend CG from post-EN-2 (t4) to Follow-up-1 (FU-1, t5) and Follow-up-2 (FU-2, t6) for arousal (left) and emotional distress (right). None of the change trajectories (multilevel regression weights) were significant. B. Graphical illustration of contrast Trend EG and Trend CG from post-ImR (t2) to post-EN-1 (t3) and post-EN-2 (t4) (left) and Trend CG, Trend EG1 and Trend EG2 from post-EN-2 (t4) to Follow-up-1 (-FU-1, t5) and Follow-up-2 (FU-2, t6) (right) for negative valence, positive valence and distress associated with negative belief. Post-ImR we found that the increase in positive valence within an ImR session (from pre-(t1) to post-ImR (t2)) decreased significantly again from t2 to t3-t4 in the EG and CG equally. None of the other change trajectories (multilevel regression weights) were significant.

**Supplementary Table 7 | Change trajectories in emotional memory characteristics: Multilevel Regression Weights in model without control variable of whether participants heard the cues**

| Contrast | Memory characteristics (primary outcomes) | | | | | Secondary outcome |
|---|---|---|---|---|---|---|
| | Negative Valence (1 – 10) | Positive Valence (1 – 10) | Arousal (1 – 10) | Vividness (1 – 10) | Emotional distress (11 – 110) | Distress negative belief (1 – 10) |
| **overall_ImR _trend** | - 0.49 (p < 0.001)*** | - 0.04 (p < 0.001)*** | - 0.12 (p < 0.001)*** | - 0.25 (p < 0.001)*** | - 0.12 (p < 0.001)*** | - 0.07 (p < 0.001)*** |
| **CG_EG** | - 0.18 | 0.02 | - 0.04 | - 0.25 | 0.002 | - 0.03 |
| **trend_CG** | 0.03 | 0.04 (p < 0.001)*** | 0.004 | 0.02 | -0.01 | 0.01 |
| **trend_EG** | 0.00 | 0.03 (p < 0.001)*** | - 0.03 (p = 0.006)** | - 0.09 (p = 0.008)** | -0.03 (p < 0.001***) | 0.01 |
| **CG1_CG2** | - 0.06 | -0.04 | 0.01 | 0.23 | 0.04 | 0.05 |
| **EG1_EG2** | 0.12 | 0.00 | 0.01 | - 0.01 | 0.07 | - 0.01 |
| **trend_EG1** | - 0.14 | 0.02 | - 0.04 | -0.26 (p = 0.003)** | - 0.02 | 0.02 |
| **trend_EG2** | - 0.06 | - 0.02 | - 0.02 | 0.02 | 0.02 | 0.04 |

Multilevel Regression Beta Weights for the 8 hypothesis-based contrasts without control variable whether participants heard something or not. Please note that due to the box-cox transformation of the outcome variable positive valence, a negative regression weight means an increase in the original positive valence variable, see Supplementary Table 1 and Figure 2.

**Supplementary Table 8 | Change trajectories in emotional memory characteristics: Multilevel Regression Weights in model with exclusion of n = 5 participants refusing the additional ENs**

| Contrast | Memory characteristics (primary outcomes) | | | | | Secondary outcome |
|---|---|---|---|---|---|---|
| | Negative Valence (1 – 10) | Positive Valence (1 – 10) | Arousal (1 – 10) | Vividness (1 – 10) | Emotional distress (11 – 110) | Distress negative belief (1 - 10) |
| **overall_ImR_trend** | -0.48 (p < 0.001)*** | - 0.04 (p < 0.001)*** | - 0.12 (p < 0.001)*** | - 0.26 (p < 0.001)*** | - 0.12 (p < 0.001)*** | - 0.07 (p < 0.001)*** |
| **CG_EG** | 0.03 | 0.03 | 0.003 | - 0.11 | 0.06 | - 0.02 |
| **trend_CG** | 0.03 | 0.04 (p < 0.001)*** | 0.004 | 0.02 | - 0.01 | 0.01 |
| **trend_EG** | 0.01 | 0.04 (p < 0.001)*** | - 0.03 (p = 0.0084)** | - 0.10 (p = 0.010915)* | - 0.03 (p = 0.00114** | 0.004 |
| **CG1_CG2** | - 0.08 | - 0.02 | 0.01 | 0.19 | 0.03 | 0.07 |
| **EG1_EG2** | 0.09 | -0.01 | 0.00 | 0.12 | 0.05 | - 0.04 |
| **trend_EG1** | - 0.14 | 0.02 | -0.04 | - 0.26 (p = 0.00372)** | - 0.02 | 0.01 |
| **trend_EG2** | 0.01 | - 0.01 | -0.02 | - 0.03 | 0.03 | 0.03 |
| **CV heard cues** | - 0.21 | 0.2 | -0.02 | -0.43 | - 0.11 | 0.24 |
| **CV heard cues:EG** | 1.24 | - 0.06 | 0.25 | 0.69 | 0.4 | - 0.02 |

Multilevel Regression Beta Weights for the eight hypothesis-based contrasts and control variable whether participants heard the cues; n = 5 participants from EG-2 were excluded from the analyses because they refused the three additional sham-EEG-nights resulting in a sample of n = 22 in EG-1 and n = 13 in EG-2. Please note that due to the box-cox transformation of the outcome variable positive valence, a negative regression weight means an increase in the original positive valence variable, see Supplementary Table 1 and Figure 2.

(A) Before the transformation

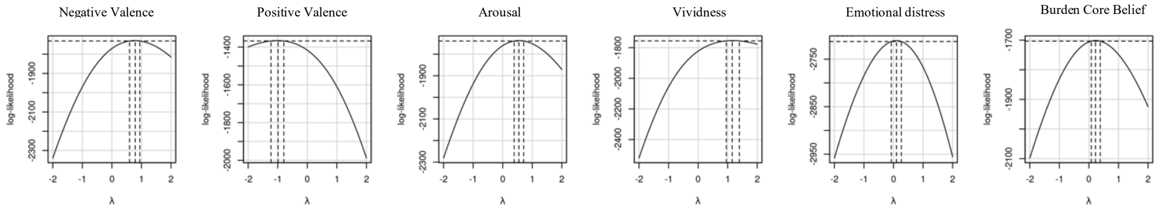


(B) After the transformation

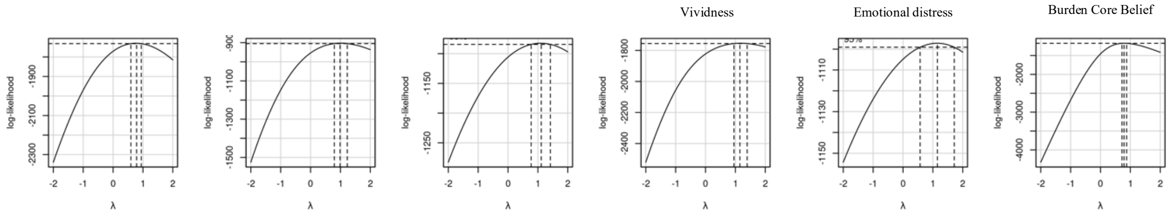


**Supplementary Figure 6 | Data pre-processing: Transformation of variables.**

Profile log-likelihoods for the parameters of the Box-Cox transformation tests. When λ = 1, no transformation is necessary; when λ = 0, a log transformation is recommended. For other values of λ, a power transformation is recommended ($y^{\lambda}$). Using the Box-Cox power transformation test (Box & Cox, 1964), we identified that 3 of the 5 key memory characteristic needed to be transformed before the analyses in order to meet the modelling assumptions, such as residuals following a Gaussian distribution. Only negative valence and vividness showed an approximately Gaussian distribution. The memory characteristic's variables arousal, EIBE sum score and distress associated with the memories negative belief showed a left-skewed distribution and were log- (arousal, distress negative belief) and square-root-transformed (EIBE) to obtain an approximately Gaussian distribution. Positive emotion showed a right-skewed distribution and we linearly transformed using a negative power transformation (y = 1/x). Please note that due to the Box-Cox transformation of the outcome variable positive valence, a negative regression weight means an increase in positive valence and vice versa, see Table 2 in the main manuscript. Follow-up Box-Cox power transformation tests confirmed the validity of the transformation of the memory characteristics variables (B).

## Supplementary Table 9 | Change trajectories in emotional memory characteristics: Multilevel Regression Weights in the model with control variables for the experimenters

| A | | | | | B | | | | |
|---|---|---|---|---|---|---|---|---|---|
| **Contrast** | **Time-points** | **TMR Condition (study group)** | **Model Expectation** | **Interpretation** | | | | | |
| | | | | | **Primary outcomes** | | | | |
| | | | | | **Negative Valence (1 – 10)** | **Positive Valence (1 – 10)** | **Arousal (1 – 10)** | **Vividness (1 – 10)** | **Emotional distress (11 – 110)** |
| **Trend overall ImR** | t1 vs. t2-t6 | same across conditions | sign. | Expected ImR main effect across the TMR conditions by comparing t1 pre-ImR against all other following time points (t2-t6) | - 0.49 *** | - 0.04*** | -0.12*** | -0.25*** | - 0.12*** |
| **Trend CG vs. EG** | across 6 time-points | CG vs. EG | not sign. | Overall condition effects across timepoints | - 0.13 | 0.04 | - 0.02 | - 0.24 | 0.01 |
| **Trend CG** | t2 vs. t3-t6 | CG only | not sign. | Expected no further change in memory characteristics after the ENs (from t3) in the CG, receiving no (CG-1) or cueing with neutral words (CG-2) | 0.03 | 0.04*** | 0.004 | 0.02 | - 0.01 |
| **Trend EG** | t2 vs. t3-t6 | EG only | sign. | Expected further improvements in memory characteristics after the ENs (from t3) in the EG receiving ImR cueing | 0.00 | 0.03*** | - 0.03** | - 0.09** | - 0.03*** |
| **Trend CG-1 vs. CG-2** | across 6 time-points | CG-1 vs. CG-2 | not sign. | Overall condition effect across timepoints | 0.03 | - 0.02 | 0.03 | 0.25 | 0.06 |
| **Trend EG-1 vs. EG-2** | across 6 time-points | EG-1 vs. EG-2 | not sign. | Overall condition effect across timepoints | 0.09 | - 0.03 | - 0.01 | - 0.02 | 0.06 |
| **Trend EG-1** | t4 vs. t5-t6 | EG-1 only | sign. | Expected improvement in memory characteristics after t4 in EG-1 receiving three additional ImR cueing nights (difference between t4 compared to t5 and t6) | - 0.14 | 0.02 | - 0.04 | - 0.26 ** | - 0.02 |
| **Trend EG-2*[1]** | t4 vs. t5-t6 | EG-2 only | not sign. | Expected no significant changes after t4 in EG-2 receiving no additional ImR cueing nights (but sham-EEG; no difference between t4 compared to t5 and t6) | - 0.06 | - 0.02 | - 0.02 | 0.02 | 0.02 |
| **CV heard cues** | across 6 time-points | EG and CG | no formulated hypothesis for CVs | Effect of the CV hearing the ImR related words | 0.62 | 0.16 * | 0.15 | 0.03 | 0.14 |
| **CV: experimenters:** | across 6 time-points | EG and CG | no formulated hypothesis for CVs | Effect of the CV controlling for the five different experimenters | | | | | |
| **Experimenter 1** | | | | | 0.48 | -0.01 | 0.02 | 0.004 | 0.02 |
| **Experimenter 2** | | | | | -0.20 | 0.03 | 0.02 | -0.41 | 0.03 |
| **Experimenter 3** | | | | | 0.06 | 0.15 | 0.10 | 0.06 | -0.06 |
| **Experimenter 4** | | | | | -0.33 | 0.06 | -0.08 | -0.20 | -0.19 |

Multilevel Regression Beta Weights for the 8 hypothesis-based contrasts with the control variables for the different experimenters. The five experimenters were included in the model as dummy-coded control variable. Please note that due to the box-cox transformation of the outcome variable positive valence, a negative regression weight means an increase in the original positive valence variable, see Supplementary Table 1 and Figure 2.

## Supplementary Table 10 | Effects of the number of cues played per experimental night on emotional memory characteristics outcomes

| Cues experimental night(s) | Memory assessment time point | F-Statistics: Effects of the number of cues played per experimental night(s) on emotional memory characteristics outcomes | | | | |
|---|---|---|---|---|---|---|
| | | **negative valence** | **positive valence** | **arousal** | **vividness** | **emotional distress** |
| experimental-night 1 (EG-1 and EG-2) | t3 | F(1, 38) = 0.03, p = 0.87 | F(1, 38) = 0.22, p = 0.65 | F(1, 38) = 1.12, p = 0.30 | F(1, 38) = 0.01, p = 0.94 | F(1, 38) = 1.11, p = 0.30 |
| experimental-night 2 (EG-1 and EG-2) | t4 | F(1, 38) = 0.73, p = 0.40 | F(1, 38) = 0.04, p = 0.85 | F(1, 38) = 1.83, p = 0.19 | F(1, 38) = 1.05, p = 0.31 | F(1, 38) = 1.53, p = 0.22 |
| additional 3 experimental nights (EG-1 only) | t5 | F(1, 38) = 0.61, p = 0.44 | F(1, 38) = 1.22, p = 0.28 | F(1, 38) = 0.37, p = 0.55 | F(1, 38) = 1.63, p = 0.21 | F(1, 38) = 2.20, p = 0.15 |
| Total number of cues across all experimental nights (EG-1 and EG-2) | t6 | F(1, 38) = 0.31, p = 0.58 | F(1, 38) = 1.31, p = 0.26 | F(1, 38) = 0.72, p = 0.40 | F(1, 38) = 0.22, p = 0.65 | F(1, 38) = 1.70, p = 0.20 |

Effects of the number of words (cues) played per experimental night on the short-term (t3 and t4) and long-term (t5 and t6) assessments of TMR outcomes in emotional memory characteristics. The results of the regression analyses (F-statistics) are presented, examining whether the number of words on the respective experimental night(s) are associated with changes in emotional memory characteristics indexed by change scores, with memory characteristics centered on their corresponding post-ImR value (tx - t2). We found no significant associations between the number of ImR cues played and the change scores in the emotional memory characteristics, both in terms of short-term effects (effects of the number of cues played in experimental nights 1 resp. 2 on the immediately following assessments at t3 resp. t4) and longer-term effects (effects of the sum of cues played in the three additional experiental nights (EG-1 only) on the assessment at t5, as well as the total number of cues played across all experimental nights on the assessment at t6). EG-1 = experimental group 1. EG-2 = experimental group 2.

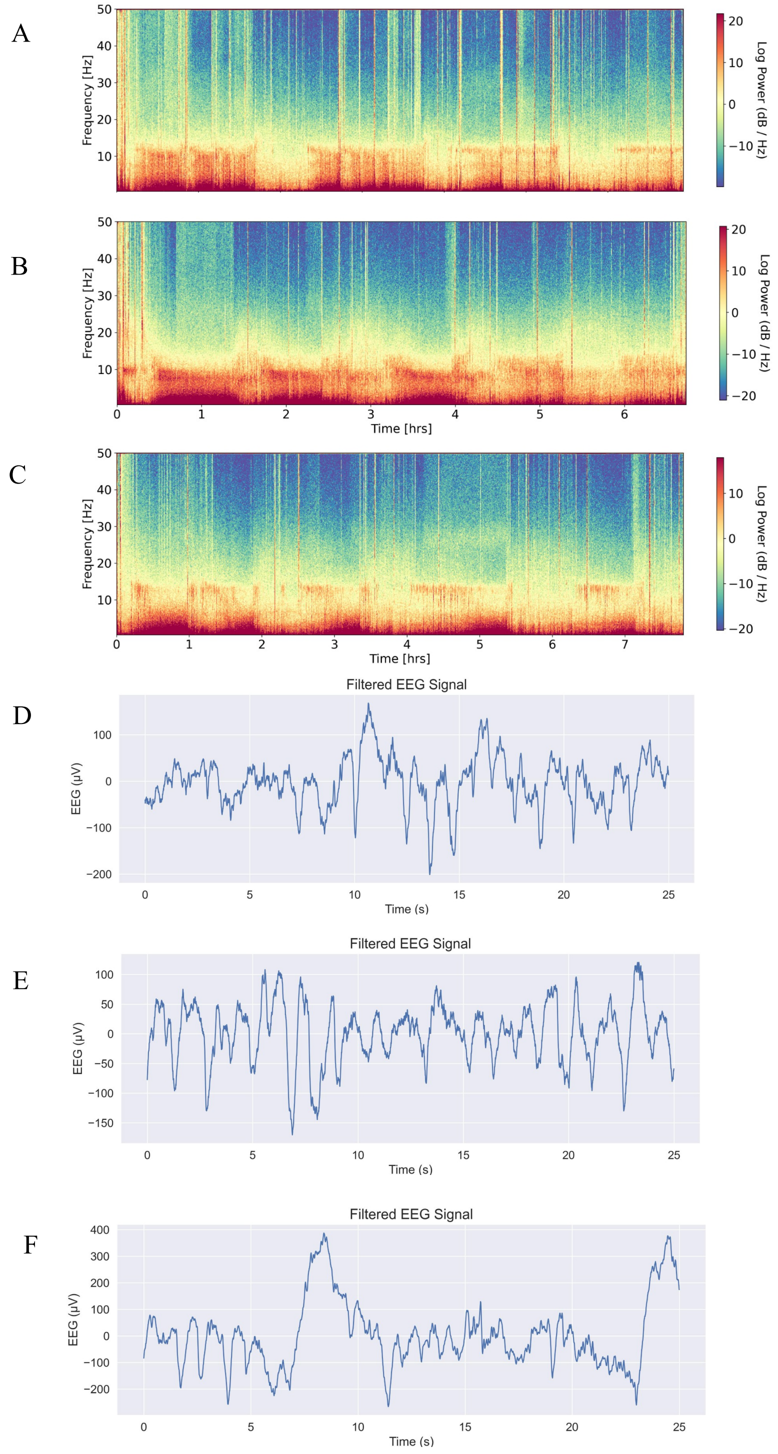


**Supplementary Figure 7 | Spectrogram and raw EEG data trace examples of MHSL-SB EEG recordings during the first experimental night of 3 randomly selected participants from the experimental group (EG).**

Plots A-C: full-night spectrograms of EEG recordings. Plots D-F: Random extract of EEG traces of 25s for each of the three EEG recordings. EEG traces are displayed after notch filtering and high pass filtering with cut-off frequency of 0.05 Hz, which are similar to the filters applied by the MHSL-SB. MHSL-SB = Mobile Health Systems Lab-Sleepband (7).

## Supplementary Table 11 | Course of emotional memory characteristics over all 6 study time points

| Time. | Total (n = 80) | EG-1 (n = 22) | EG-2 (n = 18) | CG-1 (n = 20) | CG-2 (n = 20) |
|---|---|---|---|---|---|
| **Negative valence** | | | | | |
| **T1** | 7.14 (SD = 1.44; Range: 4 – 10) | 7.14 (SD = 1.13) | 6.94 (SD = 1.47) | 7.45 (SD = 1.61) | 7 (SD = 1.59) |
| **T2** | 4.15 (SD = 1.89; Range 1-8) | 4.32 (SD = 1.91) | 4.61 (SD = 1.88) | 3.85 (SD = 1.87) | 3.85 (SD = 1.93) |
| **T3** | 4.53 (SD = 1.69; Range 1 - 9) | 4.86 (SD = 2.12) | 4.94 (SD = 1.63) | 3.95 (SD = 1.54) | 4.37 (SD = 1.21) |
| **T4** | 4.26 (SD = 1.91; Range: 1-9) | 4.82 (SD = 2.24) | 4.11 (SD = 2.19) | 4.05 (SD = 1.43) | 4 (SD = 1.65) |
| **T5** | 4.16 (SD = 1.59; Range: 1-8) | 4.45 (SD = 1.77) | 4.22 (SD = 1.77) | 3.75 (SD = 1.41) | 4.2 (SD = 1.4) |
| **T6** | 3.96 (SD = 1.80; Range: 1-9) | 4.36 (SD = 2.24) | 3.67 (SD = 1.85) | 3.7 (SD = 1.13) | 4.05 (SD = 1.82) |
| **Emotional distress (EIBE)** | | | | | |
| **T1** | 55.31 (SD = 15.97; Range: 23 – 98) | 59.18 (SD = 15.83) | 50.28 (SD = 17.32) | 58.35 (SD = 16.6 ) | 52.55 (SD = 13.41) |
| **T2** | 31.74 (SD = 17.13; Range: 11 -86) | 31.86 (SD = 18.27) | 33.89 (SD = 14.87) | 31 (SD = 20.1) | 30.4 (SD = 15.57) |
| **T3** | 30.19 (SD = 15.49; Range: 11-82) | 33 (SD = 17.39) | 28.61 (SD = 13.7) | 31 (SD = 18.17 ) | 27.58 (SD = 11.88) |
| **T4** | 28.03 (SD = 14.88; Range: 11-80) | 32.77 (SD = 18.6) | 24.06 (SD = 12.91) | 29.35 (SD = 13.52) | 25.05 (SD =12.43) |
| **T5** | 27.55 (SD = 14.09; Range: 11-74) | 29.55 (SD = 15.98) | 24.44 (SD = 10.57) | 29.95 (SD = 16.91) | 25.75 (SD = 11.59) |
| **T6** | 27.84 (SD = 12.91; Range: 11-70) | 30.32 (SD = 16.34) | 24.06 (SD = 9.1) | 29.25 (SD = 12.54) | 27.1 (SD = 11.96) |
| **Arousal** | | | | | |
| **T1** | 7.05 (SD = 1.76; Range: 2 – 10) | 7.32 (SD = 1.29) | 6.72 (SD = 1.87) | 7.35 (SD = 2.08) | 6.75 (SD = 1.8) |
| **T2** | 4.1 (SD = 2.05; Range 1-9) | 4.27 (SD = 2.35) | 4.89 (SD = 1.78) | 3.75 (SD = 1.94) | 3.55 (SD = 1.9) |
| **T3** | 4.04 (SD = 1.78; Range: 2-9) | 4.5 (SD = 2.09) | 4.11 (SD = 2) | 3.85 (SD = 1.63) | 3.63 (SD = 1.26) |
| **T4** | 3.88 (SD = 1.89; 1-9) | 4.27 (SD = 2.14) | 3.94 (SD = 2.01) | 3.45 (SD = 1.47) | 3.8 (SD = 1.88) |
| **T5** | 3.76 (SD = 1.61; Range: 1-7) | 3.77 (SD = 1.8) | 3.83 (SD = 1.5) | 3.75 (SD = 1.55) | 3.7 (SD = 1.66) |
| **T6** | 3.51 (SD = 1.63; Range: 1-8) | 3.73 2(SD = 2.16) | 3.33 (SD = 1.37) | 3.35 (SD = 1.23) | 3.6 (SD = 1.6) |
| **Positive Valence** | | | | | |
| **T1** | 1.21 (SD = 0.52; Range: 1-4) | 1.23 (SD = 0.69) | 1.33 (SD = 0.49) | 1.25 (SD = 0.55) | 1.05 (SD = 0.22) |
| **T2** | 3.06 (SD = 2.10; Range 1 - 10) | 3.23 (SD = 2.29) | 2.89 (SD = 1.75) | 3.4 (SD = 2.52) | 2.7 (SD = 1.78) |
| **T3** | 2.01 (SD = 1.49; Range: 1-8) | 1.91 (SD = 1.31) | 1.94 (SD = 1.11) | 2.45 (SD = 2.01) | 1.74 (SD = 1.37) |
| **T4** | 1.85 (SD = 1.06; Range: 1-5) | 2.14 (SD = 1.08) | 1.72 (SD = 0.96) | 2.05 (SD = 1.32) | 1.45 (SD = 0.69) |
| **T5** | 1.9 (SD = 1.14; Range: 1-6) | 2.05 (SD = 1.36) | 1.83 (SD = 0.79) | 1.9 (SD = 1.25) | 1.8 (SD = 1.11) |
| **T6** | 1.8 (SD = 1.18; Range: 1-6) | 2.14 (SD = 1.52) | 1.72 (SD = 1.02) | 1.85 (SD = 1.3) | 1.45 (SD = 0.6) |
| **Vividness** | | | | | |
| **T1** | 7.59 (SD = 1.30; Range: 5-10) | 7.64 (SD = 1.5) | 7.72 (SD = 1.13) | 7.5 (SD = 1.28) | 7.5 (SD = 1.32) |
| **T2** | 6.21 (SD = 2.03; Range 2-10) | 6.73 (SD = 2.03) | 6.72 (SD = 1.45) | 6.4 (SD = 2.21) | 5 (SD = 1.92) |
| **T3** | 6.29 (SD = 1.81; Range: 2-10) | 6.68 (SD = 1.76) | 6.44 (SD = 1.89) | 6.1 (SD = 2.1) | 5.89 (SD = 1.45) |
| **T4** | 6.1 (SD = 1.97; Range: 1-10) | 6.64 (SD = 2.01) | 6.17 (SD = 2.09) | 6 (SD = 2.2) | 5.55 (SD = 1.47) |
| **T5** | 6.03 (SD = 1.88; Range: 1-10) | 5.86 (SD = 1.64) | 6.61 (SD = 1.97) | 5.9 (SD = 2.25) | 5.8 (SD = 1.64) |
| **T6** | 5.70 (SD = 1.95: Range: 1-10) | 5.86 (SD = 2.1) | 5.83 (SD = 1.95) | 5.74 (SD = 2) | 5.35 (SD = 1.84) |
| **Distress negative belief** | | | | | |
| **T1** | 4.88 (SD = 2.25; Range: 1-9) | 4.62 (SD = 2.5) | 5.17 (SD = 2.15) | 5.32 (SD = 2.29) | 4.47 (SD = 2.06) |
| **T2** | 3.23 (SD = 1.88; Range: 1-10) | 3.38 (SD = 2.29) | 3.39 (SD = 1.91) | 3.11 (SD = 1.56) | 3.05 (SD = 1.76) |
| **T3** | 3.17 (SD = 1.71; Range: 1-9) | 3.52 (SD =1.97) | 2.94 (SD = 1.43) | 2.95 (SD = 1.61) | 3.21 (SD = 1.81) |
| **T4** | 3.12 (SD = 1.66; Range: 1-7) | 3.43 (SD = 2.01) | 3.11 (SD = 1.37) | 3.16 (SD = 1.5) | 2.75 (SD = 1.68) |
| **T5** | 3.45 (SD = 1.86; Range: 1 -8) | 3.67 (SD= 2.29) | 3.5 (SD = 1.69) | 3.26 (SD = 1.69) | 3.35 (SD = 1.79) |
| **T6** | 3.64 (SD = 2.15; Range: 1-9) | 3.82 (SD = 2.63) | 3.61 (SD = 1.91) | 3.7 (SD = 2) | 3.4 (SD = 2.06) |
| **Heart Rate (HR bpm)** | | | | | |
| **T1** | 76.35 (SD = 11.57) | 77.13 (SD = 10.76 ) | 72.37 (SD = 11.94 ) | 74.95 (SD = 7.07 ) | 82.18 (SD = 14.84 ) |
| **T2** | 72.54 (SD = 10.32) | 72.19 (SD = 7.99) | 73.38 (SD = 10.88) | 70.63 (SD = 10.74) | 74.15 (SD = 12.75) |
| **T5** | 77.15 (SD = 11.32) | 76.33 (SD = 9.18) | 75.54 (SD = 11.95) | 78.37 (SD = 10.87) | 79 (SD = 14.44) |
| **Change score (HR)** | | | | | |
| **T1** | 3.96 (SD = 7.4) | 2.15 (SD = 4.85) | 2.82 (SD = 4.46) | 3.23 (SD = 6.5) | 8.88 (SD = 11.97) |
| **T2** | 1.4 (SD = 8.95) | -2.13 (SD = 7.09) | 5.86 (SD = 8.93) | -1.41 (SD = 9.75) | 3.74 (SD = 7.96) |
| **T5** | 0.71 (SD = 6.1) | -0.86 (SD = 4.65) | 1.73 (SD = 3.74) | 2.34 (SD = 6.58) | -0.34 (SD = 9.19) |

Mean values and standard deviation (SD) of emotional memory characteristics by study group and time point.

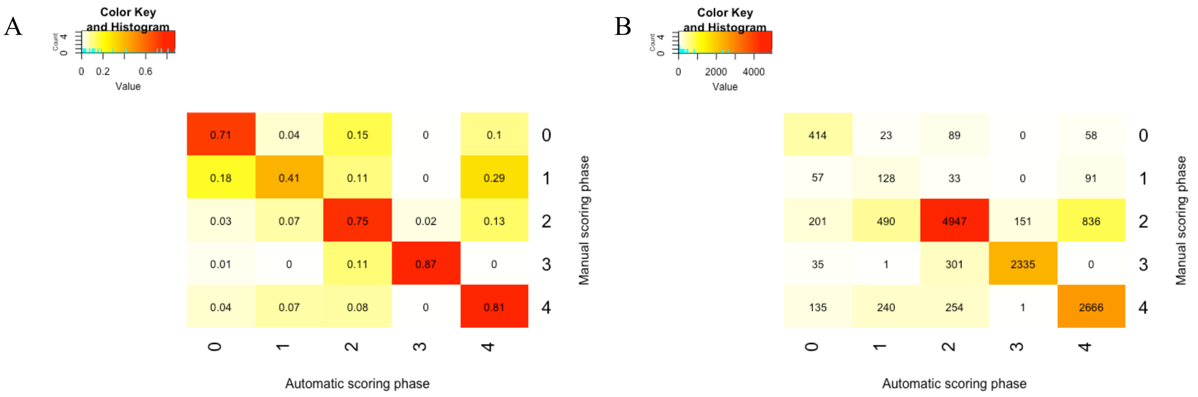


**Supplementary Figure 8 | Relative values (%) (plot A) and absolute numbers (plot B) of agreement between the automatic (deep learning algorithm-based) and expert rater sleep phase scoring.**

Manual rater scores from one expert were obtained for n = 10 representative nights, which were randomly drawn and featured the same ratio of age, sex, and time of measurement during the study period as the total sample. Depicted are the relative values (%) (plot A) and absolute numbers (plot B) of agreement between the expert rater and the automatic sleep phase scorings. Inter-rater agreement between the deep learning-based automatic sleep stage scoring algorithm and the manually scored sleep phases by the expert was adequate (Cohen's kappa = 0.68; 95% CI: 0.66 - 0.69) (8,9). 0 = wake, 1 = N1 sleep; 2 = N2 sleep; 3 = N3 sleep; 4 = REM sleep.

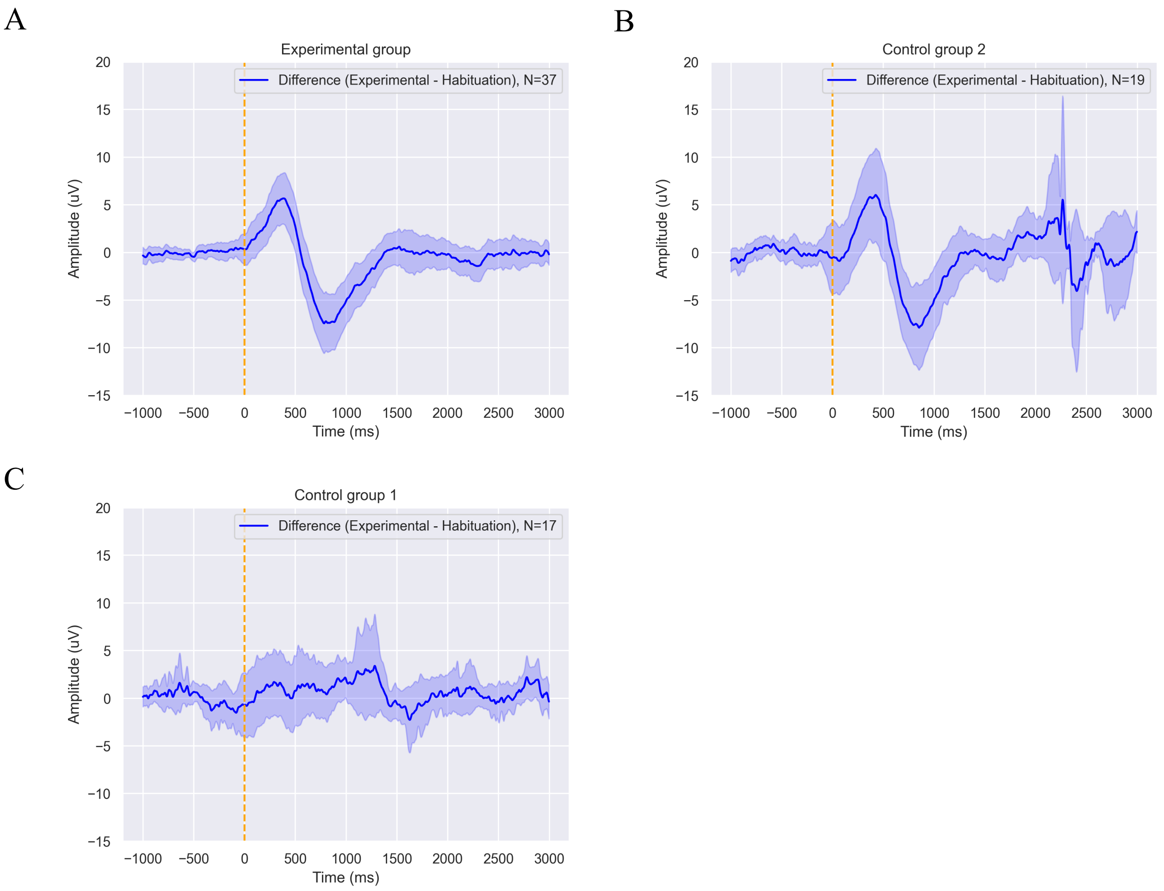


**Supplementary Figure 9 | Aggregated event-related potential (ERP) signals of TMR cue presentation.**

ERP signals after cue presentation (orange line) for the different study groups: Experimental group (EG) receiving ImR-related TMR cues (panel A), control group 2 (GC-2) receiving neutral cues (panel B), and control group 1 (CG-1) receiving no cues (panel C). Depicted are the ERPs of the experimental night subtracted from the ERPs of the second habituation night. The feedback-controlled stimulation algorithm used indicated when TMR cues were played (during experimental nights for EG and CG-2) and/or when cue presentation conditions were met according to the algorithm, but no cues were played (for all study groups during the first two habituation nights; during the experimental nights in the CG-1 receiving no TMR cues). Data was pre-processed using an established automatic artifact rejection (10). For each participant, the averaged cue-related ERP of the first experimental night was then subtracted from the averaged cue-related ERP of the second habituation night (within person). The average ERP difference (experimental night - habituation night) was then calculated for each study group (EG, CG-1, CG-2). This indicates the averaged ERPs associated with the TMR cues in the EG (plot A) and the CG-2 (plot B), both of which show averaged ERPs above 5 µV, while CG-1 (no TMR cues) shows no such increased averaged amplitudes. We chose experimental night 1 for all participants in order to exclude possible neurophysiological adaptation effects for the presentation of the TMR stimuli. For the subtraction of baseline oscillations, we selected habituation night 2, if available, as this allowed the participants to become habituated to sleeping with the wearable EEG-device (MHSL-SB) during the preceding habituation night 1. Since habituation night 2 was not recorded correctly for n = 7 participants, habituation night 1 was used for these participants. Experiment night 1 was not recorded correctly for n = 4 participants, so experiment night 2 was used instead.

A limiting factor to note is that the study used TMR cues consisting of 10 idiosyncratic words for each participant derived from individual memories. Exact temporal alignment of the cues is thus not feasible in the current ERP analysis. Despite these artifacts, we observed higher ERPs in EG and CG-2, both of which received cues on experimental night 1, compared to CG-1, which did not receive cues.

## References


1. Tukey JW. Exploratory data analysis. Vol. 2. Reading, MA; 1977.

2. Beck AT, Epstein N, Brown G, Steer RA. An inventory for measuring clinical anxiety: The Beck Anxiety Inventory. J Consult Clin Psychol,. 1988;56(6):893–7.

3. Margraf J, Ehlers A. Margraf, J., & Ehlers, A. (2007). Beck Angstinventar Deutsche Version (BAI). Bern: Hans Huber; 2007.

4. Beck AT, Steer RA, Brown GK. Manual for the Beck Depression Inventory. San Antonio: Psychological Corporation; 1996.

5. Hautzinger M, Keller F, Kühner C. Beck depressions-inventar (BDI-II). Harcourt Test Services; 2006.

6. Buysse DJ, Reynolds III CF, Monk TH, Berman SR, Kupfer DJ. The Pittsburgh Sleep Quality Index: a new instrument for psychiatric practice and research. Psychiatry research. 1989;28(2):193–213.

7. Ferster ML, Lustenberger C, Karlen W. Configurable Mobile System for Autonomous High-Quality Sleep Monitoring and Closed-Loop Acoustic Stimulation. IEEE Sensors Letters. 2019;3(5):1–4.

8. Lee YJ, Lee JY, Cho JH, Choi JH. Interrater reliability of sleep stage scoring: a meta-analysis. Journal of clinical sleep medicine : JCSM : official publication of the American Academy of Sleep Medicine. 2022;18(1):193–202.

9. Landis JR, Koch GG. The Measurement of Observer Agreement for Categorical Data. Biometrics. 1977;33(1):159.

10. Mariani S, Tarokh L, Djonlagic I, Cade BE, Morrical MG, Yaffe K, et al. Evaluation of an automated pipeline for large-scale EEG spectral analysis: the National Sleep Research Resource. Sleep Medicine. 2018 Jul;47:126–36.
